# Discovery of a selective cytochrome P450 4A inhibitor for the treatment of metabolic dysfunction‐associated fatty liver disease

**DOI:** 10.1002/ctm2.1816

**Published:** 2024-10-04

**Authors:** Minji Lee, Myung Jin Son, Sin‐Hyoung Hong, Jae‐Sung Ryu, Ji‐Hyeon Min, Dong‐Eon Lee, Ji Hoon Lee, Nam Doo Kim, Shi‐Young Park, Darong Kim, Jeongmin Joo, Jisung Kwak, Kook Hwan Kim, Yong‐Ho Lee, Byeong‐Rak Keum, Hyun Seok Song, Youngae Jung, Koon Soon Kim, Gun‐Hwa Kim

**Affiliations:** ^1^ Digital Omics Research Center Korea Basic Science Institute (KBSI) Cheongju Republic of Korea; ^2^ Stem Cell Convergence Research Center Korea Research Institute of Bioscience and Biotechnology (KRIBB) Daejeon Republic of Korea; ^3^ Department of Functional Genomics Korea University of Science & Technology (UST) Daejeon Republic of Korea; ^4^ School of Pharmacy Sungkyunkwan University Suwon Republic of Korea; ^5^ New Drug Development Center Osong Medical Innovation Foundation Cheongju Republic of Korea; ^6^ Department of Analytical Science and Technology Graduate School of Analytical Science and Technology (GRAST) Chungnam National University Daejeon Republic of Korea; ^7^ Department of Bio‐Analytical Science University of Science and Technology (UST) Daejeon Republic of Korea; ^8^ New Drug Development Center Daegu‐Gyeongbuk Medical Innovation Foundation Daegu Republic of Korea; ^9^ New Drug Discovery & Development VORONOIBIO Inc. Incheon Republic of Korea; ^10^ Korea Mouse Metabolic Phenotyping Center Lee Gil Ya Cancer and Diabetes Institute Gachon University Incheon South Korea; ^11^ Sensor System Research Center Korea Institute of Science and Technology (KIST) Seoul Republic of Korea; ^12^ Department of Chemical and Biological Engineering Korea University Seoul Republic of Korea; ^13^ Discovery Division GI Innovation, Inc. Seoul South Korea; ^14^ Department of Internal Medicine Yonsei University College of Medicine Seoul South Korea; ^15^ Department of Life Sciences Pohang University of Science and Technology Pohang Republic of Korea; ^16^ Integrated Metabolomics Research Group Metropolitan Seoul Center Korea Basic Science Institute (KBSI) Seoul Republic of Korea; ^17^ Division of Endocrinology and Metabolism Daejeon Endo Internal Medicine Daejeon Republic of Korea; ^18^ Research and Development Center CYPHARMA Daejeon Republic of Korea

Dear Editor,

Metabolic dysfunction‐associated fatty liver disease (MAFLD), a revised definition of nonalcoholic fatty liver disease (NAFLD), comprises patients with hepatic steatosis who fulfil the criteria of overweight/obesity, type II diabetes mellitus (T2DM), or more than two metabolic abnormalities,^1^ providing a valuable tool for identifying patients with fatty liver at higher risk of disease progression.^2^ MAFLD is a complex disease in which various pathogenic factors contribute to its progression, including fat accumulation, lipotoxicity, oxidative stress, and endoplasmic reticulum (ER) stress. The heterogeneous risk profile of MAFLD presents challenges for effective treatment. Within the mammalian liver, cytochrome P450 4A (CYP4A) functions as a fatty acid hydroxylase, actively participating in oxidative metabolism and catalyzing the breakdown of lipid peroxides, consequently generating reactive oxygen species (ROS). Previous research has demonstrated that targeting CYP4A shows potential in exploring the pathophysiology of liver diseases, including MAFLD and diabetes.^3^, ^4^, ^5^ Taking these findings together, we suggest that CYP4A holds significant promise for the treatment of MAFLD, and the discovery of a CYP4A inhibitor may serve as a potent drug candidate.

We identified CYP4A inhibitors through in silico analysis. Among several hit compounds, C418 significantly reduced CYP4A enzyme activity. Subsequently, we synthesized derivatives, namely C4181 (C1) and C4182 (C2) (Figure 1A). Both C1 and C2 exhibited substantial reduction at a dose of 5 µM (Figures S1A and S2A), demonstrating potent inhibition with IC_50_ (Figure 1B). Importantly, neither compound exhibited cytotoxic effects (Figure S1B,C) and demonstrated drug‐like properties in various assays, including CYP inhibition, metabolic stability, plasma stability, and permeability (Tables S1 and S2).

FIGURE 1Characterization of the novel potent C418 derivatives (C4181 and C4182). (A) Homology model structure of cytochrome P450 4A (CYP4A) with C4181(C1) or C4182 (C2) using glide‐based docking strategy. (B) Normalized CYP4A enzyme activity in the presence of vehicle (VE), HET0016 (HET), or CYP4A inhibitors (C1 or C2) and Mean IC_50_ values. (C–L) C57BL/6N mice were fed a normal chow diet (NCD) or high‐fat diet (HFD) and administered with either vehicle (VE) or CYP4A inhibitors (C1 or C2, 5 mg/kg/day) intraperitoneally for 12 weeks. (C) Scheme of the C1 or C2 administration to HFD‐induced diabetic mice. (D) Body masses and food intake of each mice group. (E) Glucose tolerance testing (GTT) and insulin tolerance testing (ITT) data. (F) Isolated liver tissues (scale bar = 10 mm) and representative H&E‐stained liver sections (scale bar = 100 µm). (G) Hepatic triglyceride (TG) concentration. (H) Quantification of CD36 (Fatty acid translocase) protein levels normalized to β‐actin. (I) Lipid peroxidation, assessed by the measurement of malondialdehyde (MDA) concentration. (J) Hepatic 20‐HETE concentration. (K, L) Quantification of western blot. Relative protein levels normalized to β‐actin and phosphorylated protein levels normalized to total protein Western blots of C1 and C2 on the gluconeogenesis, lipogenesis, ER stress, insulin resistance, and apoptosis of HFD‐fed mice. All experiments were performed as multiple independent samples, and the values are shown as mean ± SEM, analyzed by the Student's *t*‐test or two‐way ANOVA. *n *= 3‐5; *p < .05, **p < .01, *** < .001 for NCD + VE versus HFD + VE versus HFD + C1 or C2. ns, not significant.

**Figure:**
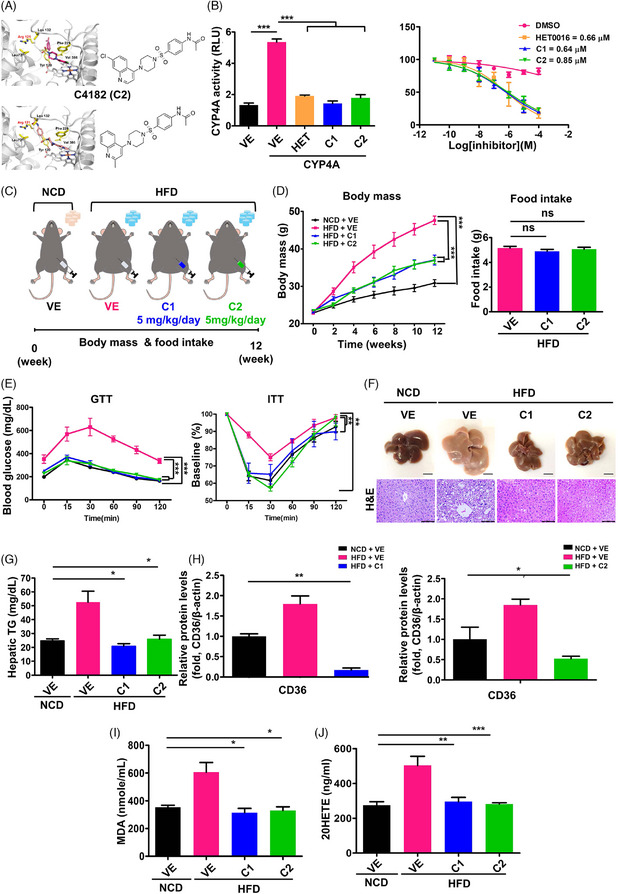

**Figure:**
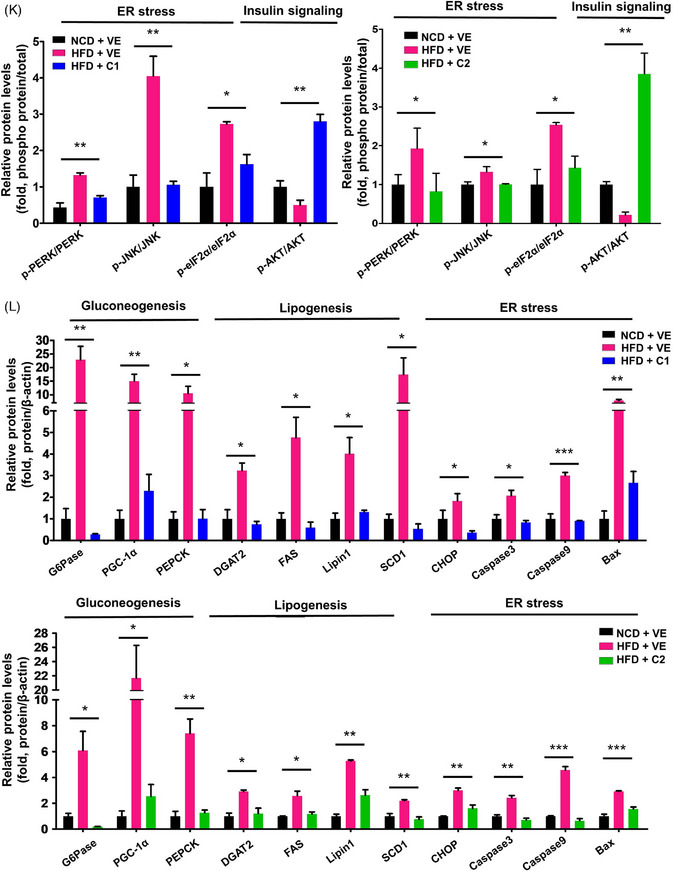

We investigated the effects of novel CYP4A inhibitors using HepG2 cells exposed to lipid overload or induced ER stress. C1 and C2 increased glucose uptake (Figures S1D and S2B) and significantly mitigated lipid accumulation and ROS generation (Figures S1E,F and S2C,D). Furthermore, they restored the expression of proteins implicated in gluconeogenesis, lipogenesis, and ER stress (Figures S1G‐K and S2E), indicating the effectiveness of CYP4A inhibitors in in vitro models. Additionally, we determine the effect of C1 and C2 in various in vivo models, encompassing diet‐induced T2DM, *db/db* mice, and metabolic dysfunction‐associated steatohepatitis (MASH). In the diet‐induced T2DM model, we determined the optimal concentration of C1 and C2 (Figure S3) and observed a significant reduction in body mass without affecting food intake (Figure 1C,D). Glucose tolerance and insulin tolerance testing (ITT) exhibited significant improvements (Figure 1E), and serum glucose and insulin concentrations were abrogated (Table S3). Histological analysis revealed improved hepatic steatosis, with significant reductions in both hepatic and serum triglyceride (TG) concentrations and CD36 expression (Figure 1F–H, Figure S4A and Table S3). Moreover, C1 and C2 treatment decreased the concentration of hepatic malondialdehyde (Figure 1I) and 20‐HETE, which is generated as a product of CYP4A enzymatic action (Figure 1J).^6^ The treatments also improved serum concentrations of aspartate aminotransferase (AST), alanine transaminase (ALT), total cholesterol, LDL‐cholesterol, LDL/HDL‐cholesterol ratio and adiponectin (Table S3). Notably, the expression of mediators of gluconeogenesis, lipogenesis, ER stress, apoptosis, and insulin resistance was significantly restored (Figure 1K,L and Figure S4B,C). We next assessed the effects of C1 and C2 administration in *db/db* mice, which showed improvements in metabolic defects, ER stress, apoptosis, and insulin resistance, consistent with the effects observed in the diet‐induced T2DM model (Figure S5).

Finally, we assessed the effect of C1 and C2 in the MASH model, induced by a methionine‐choline‐deficient (MCD) diet using *ob/ob* mice to replicate MASH histology (Figure 2A).^7^ Although the MCD diet resulted in reductions in body weight over time (Figure S6), the CYP4A inhibitors showed significant improvement effects on MASH. They significantly improved serum glucose concentrations (Figure 2B), as well as both their serum and hepatic TG concentrations (Figure 2C,D). Histological examination demonstrated a significant reduction in intracytoplasmic lipid accumulation and collagen deposition (Figure 2E). Additionally, there was a decrease in hepatic 20‐HETE concentration (Figure 2F), and serum AST and ALT activities were nearly normalized (Figure 2G), suggesting that C1 and C2 alleviate hepatic steatosis and liver damage. Furthermore, the expression of genes related to hepatic inflammation and fibrosis was significantly lower in the treated mice (Figure 2H,I). Therefore, C1 and C2 ameliorate steatosis and MASH.

**FIGURE 2:**
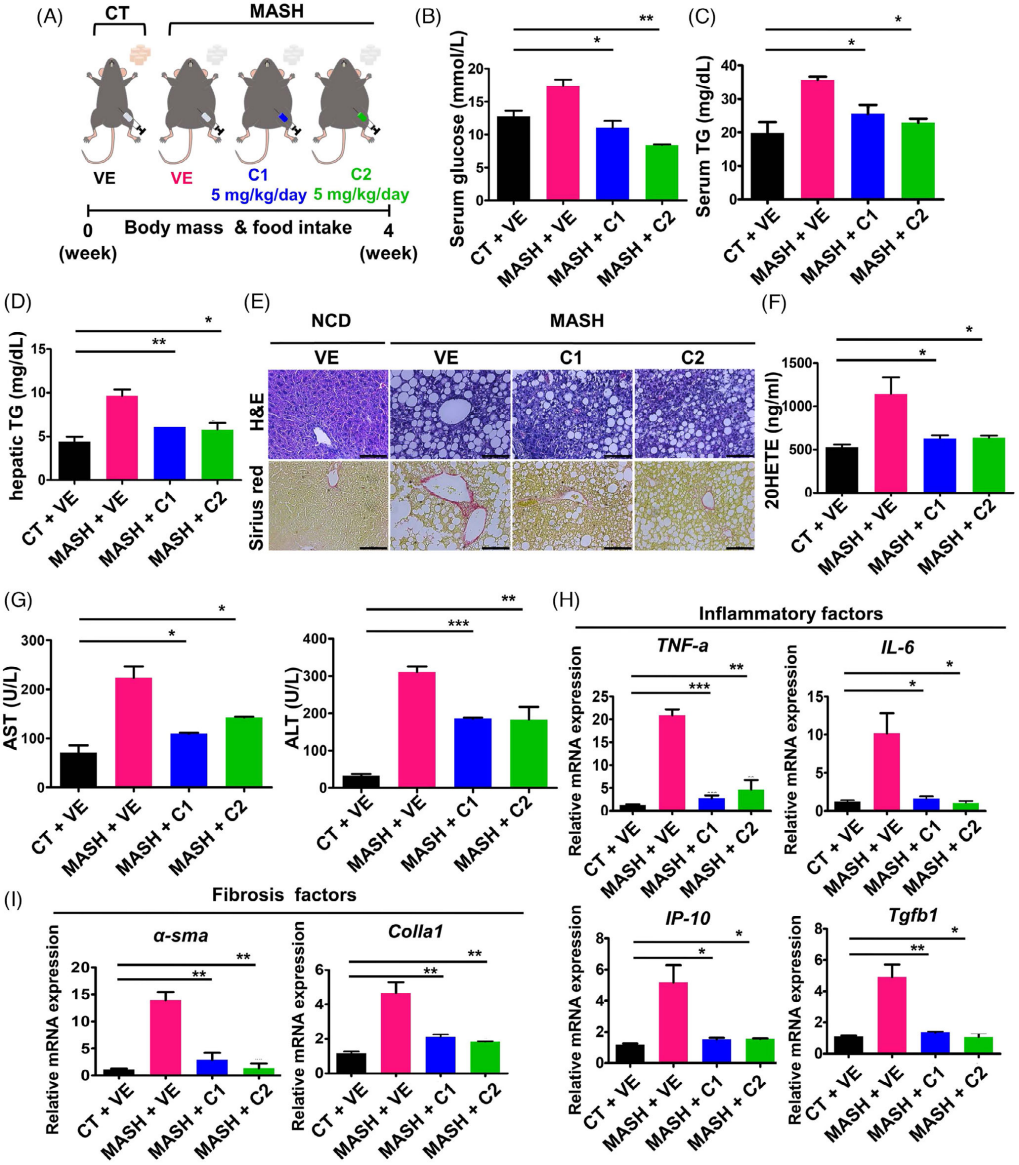
Effects of C1 and C2 in metabolic dysfunction‐associated steatohepatitis (MASH) model. C57BL/6N and MASH model, respectively, and administered either vehicle (VE) or one of the candidate drugs (C1 or C2, 5 mg/kg/day) intraperitoneally for 4 weeks. (A) Scheme for the experiment. (B) Serum glucose concentration. (C) Serum triglyceride (TG) concentration. (D) Hepatic triglyceride concentration. (E) Representative liver sections stained with H&E (intracytoplasmic lipid accumulation, *top*) or Sirius red (collagen deposition*, bottom*) (scale bar = 100 µm). (F) Hepatic 20‐HETE concentration. (G) Serum AST and ALT activities. (H, I) mRNA expression of genes mediating inflammation and fibrosis. All experiments were performed as multiple independent samples, and the values are shown as mean ± SEM, analyzed by the two‐way ANOVA. *n *= 3; *p < .05, **p < .01 and ***p < .001 for CT (CT; control mice) + VE versus MASH + VE versus MASH + C1 or C2.

Recently, it has been reported that patients with MAFLD,^5^, ^8^ exhibit elevated expression of CYP4A in the liver, as was also observed in Korean patients (Figure S7). However, investigating the direct effect of CYP4A inhibitors in humans is challenging. To overcome this limitation, three‐dimensional models were utilized as effective tools for preclinical drug discovery. We employed established models, including organoid‐based and 3D HepaRG models of liver steatosis.^9^, ^10^ Treatment with compounds C1 and C2 notably decreased the elevated mRNA expression and activity of CYP4A (Figure 3A,B and Figure S8A,B), improved glucose uptake and consumption (Figure 3C–E and Figure S8C), reduced lipid accumulation (Figure 3F–H and Figure S8D) and lowered triglyceride levels (Figure 3I and Figure S8E). Moreover, the expression of CD36 (Figure 3J) and various mediators related to lipogenesis, gluconeogenesis, ER stress and insulin resistance were downregulated by C1 and C2 in both organoid‐based (Figure 3K–N and Figure S9) and 3D HepaRG models (Figure S8F,G).

**FIGURE 3:**
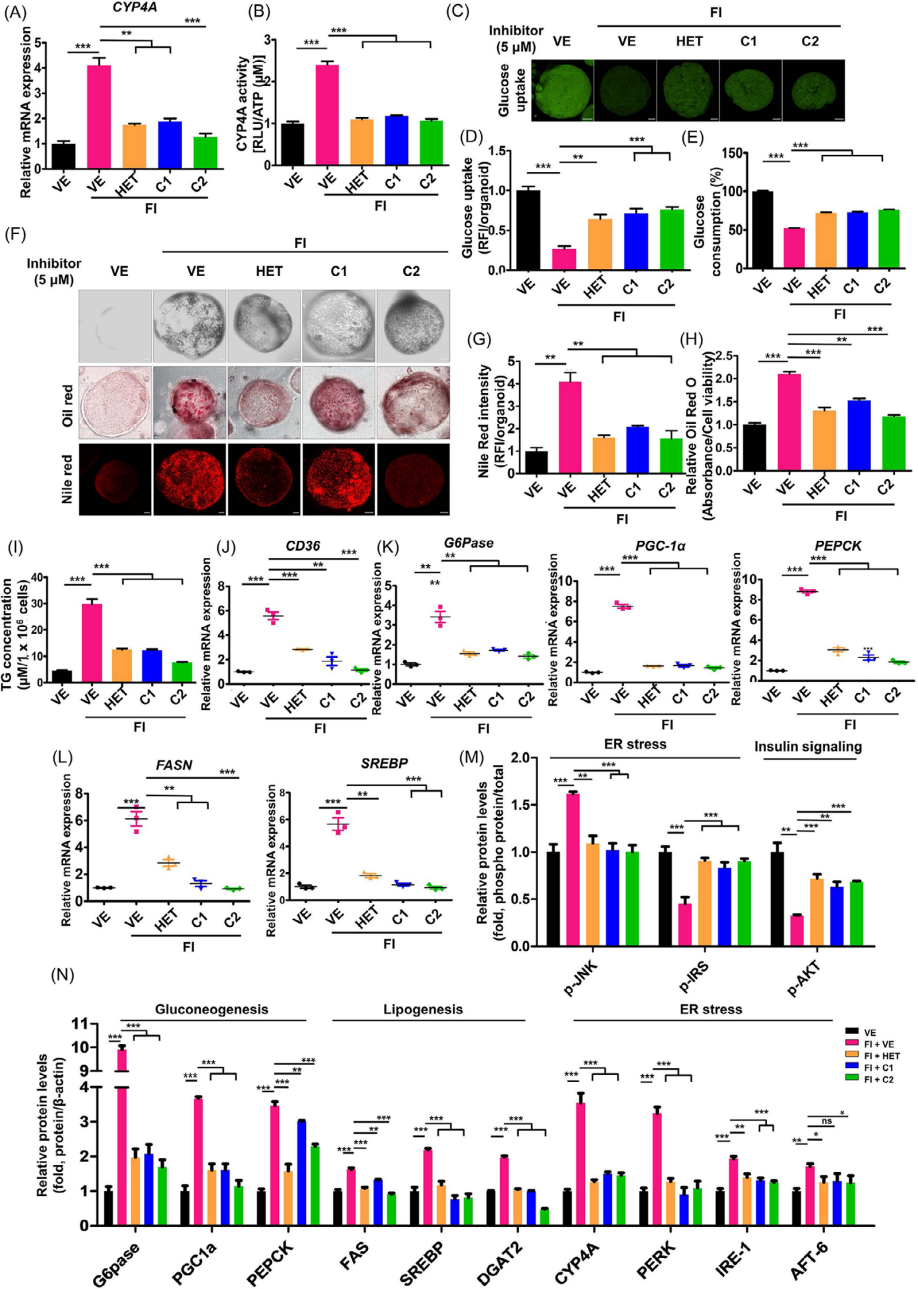
Effects of C1 and C2 in a 3D organoid‐based model of liver steatosis. (A) Normalized mRNA expression of cytochrome P450 4A (CYP4A) after treatment with vehicle (VE), HET0016 (HET) or CYP4A inhibitors (C1 or C2) for 3 days in an organoid model of 0.5 mM oleate and 0.25 mM palmitate‐induced liver steatosis (Fatty Induction, FI). (B) CYP4A activity under each condition. (C) Glucose uptake over 4 h, was assessed using a fluorescent glucose analogue (scale bar = 50 µm). (D) Normalized glucose uptake. (E) Normalized glucose consumption over 48 hours. (F) Representative cell morphology (*upper*) and fluorescence images of organoids stained with Oil red O (*middle*) and Nile Red (*lower*) (scale bar = 50 µm). (G) Normalized intensity of Nile Red staining. (H) Normalized intensity of Oil red O staining. (I) Organoid triglyceride (TG) concentration. (J) mRNA expression of CD36. (K) mRNA expression of genes involved in gluconeogenesis. (L) mRNA expression of genes involved in lipogenesis. (M, N) Quantification of western blot. (M) Relative protein levels normalized to β‐actin. (N) Phosphorylated protein Levels normalized to total protein. Western blots for expression markers involved in gluconeogenesis, lipogenesis, ER stress, and insulin signalling. All experiments were performed as multiple independent samples, and the values are shown as mean ± SEM, analyzed by the Student's *t*‐test. *n *= 3; **p < .01 and ***p < .001 for VE versus FI + VE and FI + VE versus FI + HET or C1 or C2.

To understand the mechanism of C1 and C2, we conducted transcriptomic analysis, focusing on differentially expressed genes and pathway analysis (Figure S10A–D and Table S4). Utilizing Ingenuity Pathway Analysis and Gene Set Enrichment Analysis, we observed a significant reduction in the expression of genes associated with hepatic steatosis, inflammation, T2DM, and MAFLD upon treatment with C1 and C2 (Figure 4A–D). Transcriptomic profiling further indicated a decrease in inflammation and ER stress‐related genes following treatment with the CYP4A inhibitors (Figure 4E,F and Figure S10E,F). C1 and C2 also present similar downregulated genes related to MAFLD (Table S5). Collectively, these data demonstrate that inhibiting CYP4A effectively improves MAFLD by reducing hepatic steatosis, inflammation, and fibrosis, while enhancing ITT.

FIGURE 4Comparison of transcriptomic profiles of high‐fat diet (HFD)‐fed mice and HFD‐fed mice treated with C1 or C2. (A–D) Core analysis using Ingenuity Pathway Analysis (IPA) and Gene Set Enrichment Analysis (GSEA) in HFD compared with HFD + C1 or HFD compared with HFD + C2 were carried out. The molecular network and pathway analysis showed that C1 and C2 considerably reduced the expression of genes related to hepatic steatosis, inflammation, type II diabetes mellitus (T2DM) and metabolic dysfunction‐associated fatty liver disease (MAFLD). (A) Core analysis of HFD compared with HFD treated with C1. (B) Core analysis of HFD compared with HFD treated with C2. (C) Gene Set Enrichment Analysis of C1. (D) Gene Set Enrichment Analysis of C2. (E, F) Functional enrichment analysis was performed using comparison analysis of IPA. (E) Key regulatory networks of C1 related to inflammation of the liver, liver damage and hepatic injury. (F) Key regulatory networks of C2 related to hepatic lipid metabolism and ER stress. Red indicates upregulated genes, and green indicates downregulated genes. Orange represents the predicted activation of nodes, and blue represents the predicted inactivation of nodes. (G) Schematic illustrating the effects of cytochrome P450 4A (CYP4A) inhibitors in the livers of individuals with MAFLD through three different mechanisms; ER stress/oxidative stress, Fatty acid translocase (FAT/CD36)/Lipotoxicity, and inflammation/fibrosis.

**Figure:**
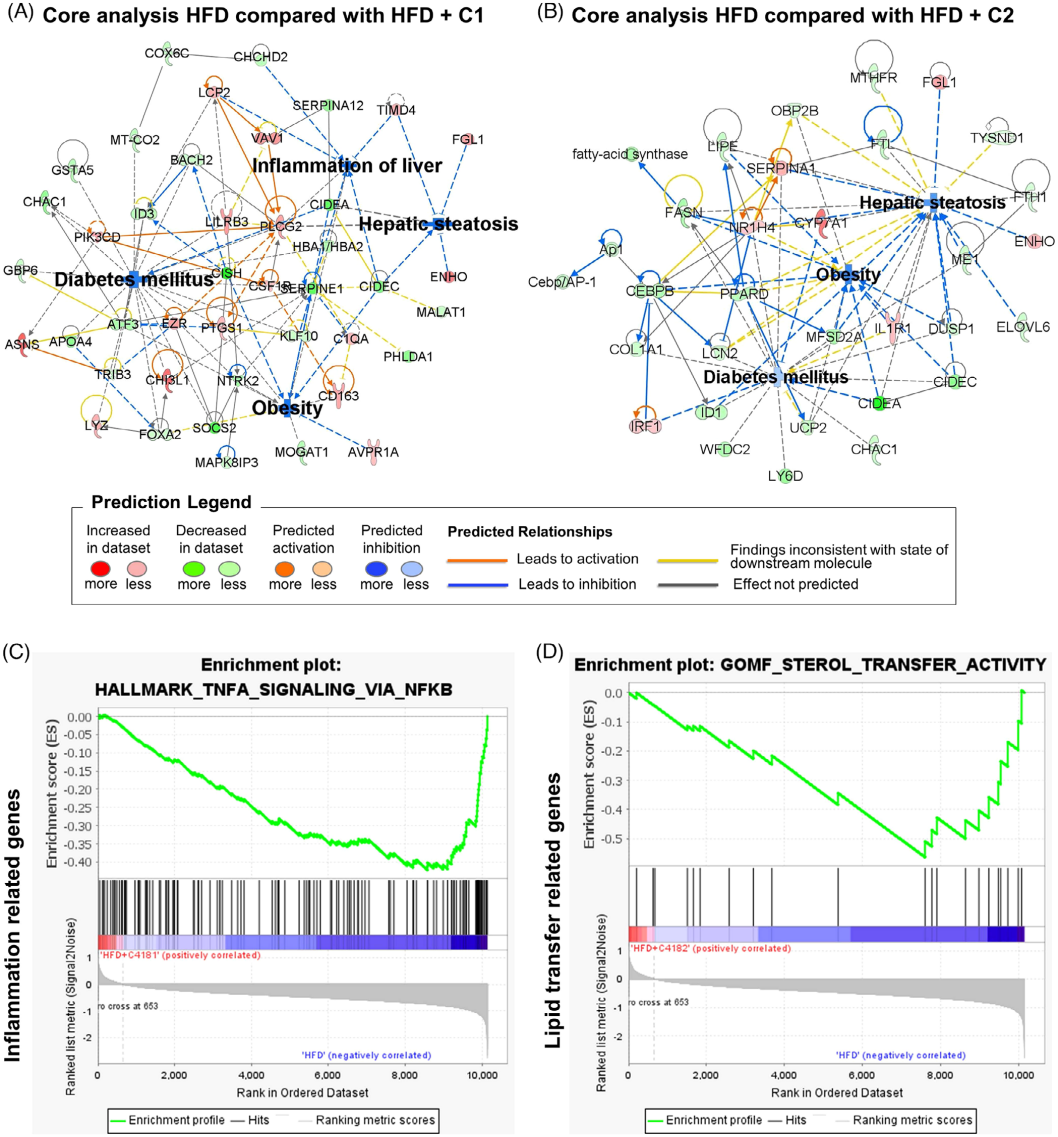

**Figure:**
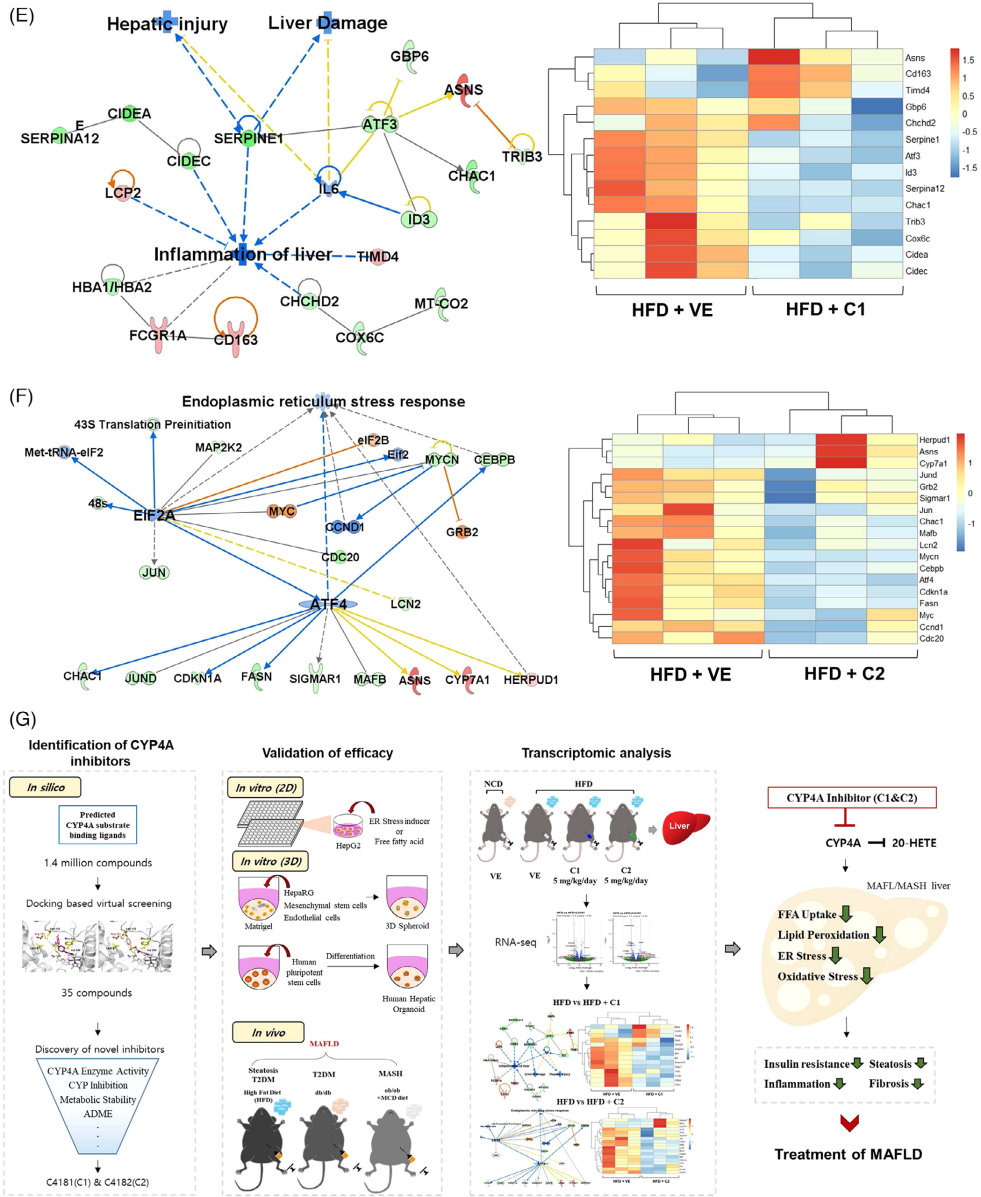

In conclusion, our findings highlight the promising therapeutic potential of CYP4A inhibitors in addressing MAFLD. These inhibitors operate through three distinct mechanisms: ER stress/oxidative stress, Fatty acid translocase (FAT/CD36)/lipotoxicity, and inflammation/fibrosis (Figure 4G). Our results demonstrate that these inhibitors could serve as innovative candidates, introducing a novel concept in the medical field for the treatment of MAFLD.

## AUTHOR CONTRIBUTIONS

Minji Lee, Myung Jin Son and Sin‐Hyoung Hong contributed equally to this work. Conceptualization: Minji Lee, Myung Jin Son and Gun‐Hwa Kim; Methodology: Sin‐Hyoung Hong, Jisung Kwak, Kook Hwan Kim, Yong‐Ho Lee and Hyun Seok Song; Investigation: Minji Lee, Myung Jin Son, Sin‐Hyoung Hong, Jae‐Sung Ryu, Ji‐Hyeon Min and Dong‐Eon Lee; Visualization: Minji Lee, Myung Jin Son and Byeong‐Rak Keum; Formal analysis: Minji Lee, Myung Jin Son, Ji Hoon Lee, Nam Doo Kim, Shi‐Young Park, Darong Kim and Jeongmin Joo, Youngae Jung; Resources: Ji Hoon Lee, Nam Doo Kim, Darong Kim and Jeongmin Joo; Funding acquisition: Gun‐Hwa Kim; Project administration: Koon Soon Kim and Gun‐Hwa Kim; Supervision: Gun‐Hwa Kim; Writing—original draft: Minji Lee; Writing—review & editing: Gun‐Hwa Kim. All authors have read and approved the article.

## CONFLICT OF INTEREST STATEMENT

The authors declare no conflict of interest.

## FUNDING INFORMATION

This work was supported by grants from the Korea Basic Science Institute (Grant number C270300) and the National Research Foundation of Korea (Grant number NRF‐2021R1A2C1008663).

## Supporting information

Supporting Information

## Data Availability

The datasets of RNA sequencing in the present study are available in the GEO database under the accession number GSE229697. All data supporting the findings of this study are available from the corresponding author upon reasonable request.

## References

[1] Eslam M , Newsome PN , Sarin SK , et al. A new definition for metabolic dysfunction‐associated fatty liver disease: an international expert consensus statement. Journal of Hepatology. 2020;73(1):202‐209. 32278004 10.1016/j.jhep.2020.03.039

[2] Eslam M , Sanyal AJ , George J , et al. MAFLD: a consensus‐driven proposed nomenclature for metabolic associated fatty liver disease. Gastroenterology. 2020;158(7):1999‐2014. e1. 32044314 10.1053/j.gastro.2019.11.312

[3] Park EC , Kim SI , Hong Y , et al. Inhibition of CYP4A reduces hepatic endoplasmic reticulum stress and features of diabetes in mice. Gastroenterology. 2014;147(4):860‐869. doi:10.1053/j.gastro.2014.06.039 24983671

[4] Lin JH , Li H , Yasumura D , et al. IRE1 signaling affects cell fate during the unfolded protein response. Science. 2007;318(5852):944‐949. 17991856 10.1126/science.1146361 PMC3670588

[5] Zhang X , Li S , Zhou Y , et al. Ablation of cytochrome P450 omega‐hydroxylase 4A14 gene attenuates hepatic steatosis and fibrosis. Proc Natl Acad Sci U S A. 2017;114(12):3181‐3185. doi:10.1073/pnas.1700172114 28270609 PMC5373383

[6] Edson KZ , Rettie AE . CYP4 enzymes as potential drug targets: focus on enzyme multiplicity, inducers and inhibitors, and therapeutic modulation of 20‐hydroxyeicosatetraenoic acid (20‐HETE) synthase and fatty acid ω‐hydroxylase activities. Curr Top Med Chem. 2013;13(12):1429‐1440. 23688133 10.2174/15680266113139990110 PMC4245146

[7] Li H , Toth E , Cherrington NJ . Asking the right questions with animal models: methionine‐ and choline‐deficient model in predicting adverse drug reactions in human NASH. Toxicol Sci. 2018;161(1):23‐33. doi:10.1093/toxsci/kfx253 29145614 PMC6454421

[8] Gao H , Cao Y , Xia H , Zhu X , Jin Y . CYP4A11 is involved in the development of nonalcoholic fatty liver disease via ROS‐induced lipid peroxidation and inflammation. Int J Mol Med. 2020;45(4):1121‐1129. doi:10.3892/ijmm.2020.4479 32124935 PMC7053872

[9] Ryu JS , Lee M , Mun SJ , et al. Targeting CYP4A attenuates hepatic steatosis in a novel multicellular organotypic liver model. J Biol Eng. 2019;13:69. doi:10.1186/s13036-019-0198-8 31406506 PMC6686528

[10] Mun SJ , Ryu JS , Lee MO , et al. Generation of expandable human pluripotent stem cell‐derived hepatocyte‐like liver organoids. J Hepatol. 2019;71(5):970‐985. doi:10.1016/j.jhep.2019.06.030 31299272

