# Supplementary material for: Discovery of a selective cytochrome P450 4A inhibitor for the treatment of metabolic dysfunction‐associated fatty liver disease
Source: Clin Transl Med. 2024 Oct 4;14(10):e1816. doi: 10.1002/ctm2.1816 (PMC11452733; doi:10.1002/ctm2.1816)
Supplement: Supplementary file 1 — Supporting Information [file CTM2-14-e1816-s001.docx]

**Supporting Information**

**Discovery of a Selective CYP4A Inhibitor for the Treatment of Metabolic Dysfunction-Associated Fatty Liver Disease (MAFLD)**

Minji Lee ^1,†^, Myung Jin Son ^2,3,4,†^, Sin-hyoung Hong ^1,†^, Jae-Sung Ryu ^5^, Ji-hyeon Min ^1,6^, Dong-eon Lee ^1,7^, Ji Hoon Lee ^8^, Nam Doo Kim ^9^, Shi-Young Park ^10^, Darong Kim ^8^, Jeongmin Joo ^8^, Jisung Kwak ^11,12^, Kook Hwan Kim ^13^, Yong-Ho Lee ^14^, Byeong-Rak Keum ^15^, Hyun Seok Song ^11^, Youngae Jung ^16^, Koon Soon Kim ^17^, Gun-Hwa Kim ^1,6,7,18,*^

**Materials and Methods**

**Table S1**

**Table S2**

**Table S3**

**Table S4**

**Figure S1**

**Figure S2**

**Figure S3**

**Figure S4**

**Figure S5**

**Figure S6**

**Figure S7**

**Figure S8**

**Figure S9**

**Figure S10**

**Materials and Methods**

**Homology Model Structure of CYP4A**

As there is no crystal structure of CYP4A, we built a homology model structure of CYP4A. For the prediction of CYP4A model structure, we used the crystal structure of the CYP3A4 from the Protein Data Bank (PDB code: 3UA1). The sequence of CYP4A was retrieved from The UniProt Database. Sequence alignment of CYP4A and template protein structure was generated using the Discovery Studio 2016. The overall sequence identities between CYP4A and the CYP3A4 are 40%. The homology model structure of CYP4A was built by using the Modeller in Discovery Studio 2016 package.

**Ligand Preparation and Virtual Screening**

To identify inhibitors against the CYP4A substrate binding site, high throughput virtual screening was performed using the commercially available ChemDiv’s compounds database. To achieve this objective, we employed Schrodinger's LigPrep tool to generate three-dimensional conformers for a preliminary dataset of 1.4 million compounds that were initially represented in two-dimensional structures. The compounds were subjected to Glide based docking strategy in which all the compounds were docked by three stages of the docking protocol, High Throughput Virtual Screening (HTVS), Standard Precision (SP), and Extra Precision (XP). A receptor grid was generated using the Receptor Grid Generation protocol with a centroid at the CYP4A heme binding site. A scaling factor of 0.7 was set to *van der Waals* radii for the atoms of residues that presumably interact with ligands. No further modifications were applied to the default settings. The final 35 compounds were selected based on the Glide score function.

**Cytochrome P450 inhibition assay**

The incubation mixtures contained 0.25 mg/mL pooled human liver microsome, 0.1M phosphate buffer (pH 7.4), and various P450 enzyme-specific substrate cocktails (Phenacetin 50 μM, diclofenac 10 μM, S-mephenytoin 100 μM, dextromethorphan 5 μM, midazolam 2.5 μM), and HET0016 or CYP4A inhibitors serially diluted were pre-incubation for 5 min at 37 ℃. The reaction was initiated by the addition of nicotinamide adenine dinucleotide phosphate (NADPH) regenerating system incubation and performed at 37 ℃ for 15 min in a thermo mixer. The reaction was stopped by the addition of ice-cold acetonitrile containing terfenadine as an internal standard. The incubation mixtures were then centrifuged at 14,000 × g for 5 min at 4 °C, and the supernatant was analyzed by LC-MS/MS system. The five CYP450 metabolites were analyzed using a Thermo Vantage Triple quadrupole mass spectrometer (Thermo Fisher Scientific Inc., MA, USA) and Shimadzu Nexera XR system. The HPLC column was a Kinetex C18 column (2.1 × 100 mm^2^, 2.6 µm particle size; Phenomenex, CA, USA). The mobile phase consisted of 0.1% formic acid in water and 0.1% formic acid in acetonitrile, and a gradient program was used. Quantitation was performed by using SRM (Selected Reaction Monitoring) modes, and data were processed by Xcalibur software (version 1.6.1).

**Measurement of Microsomal Stability**

To evaluate metabolic stability assay, human or rat liver microsomes (0.5 mg/mL), potassium phosphate buffer (0.1 M, pH 7.4) and test compound (1 μM) were pre-incubated 5 min at 37 ℃. The reaction was started by the addition of NADPH regeneration system and incubated for 0 and 30 min in a thermo mixer. The reaction was quenched by the addition of ice-cold acetonitrile containing chlorpropamide as an internal standard. Precipitated proteins were removed by centrifugation at 14,000 × g for 5 min at 4 °C. Aliquots of the supernatant were injected onto an LC-MS/MS system. Percent of the parent compound remaining was calculated by comparing the peak area ratio at 0 min and 30 min.

**Measurement of Plasma Stability**

The plasma stability assay was conducted by incubating a test compound with either human or rat plasma for 120 min at 37 °C^1^. After the incubation period, the reaction was stopped by adding ice-cold acetonitrile and vortexing the samples for 5 min. The precipitated proteins were then separated from the supernatant by centrifugation at 14,000 × g for 5 minutes at 4 °C. Aliquots of the resulting supernatant were injected onto a Shimadzu Nexera XR LC system. The percentage of the parent compound remaining in the plasma samples was determined by comparing peak areas using the Xcalibur software (version 1.6.1, Waltham, MA, USA)

**Parallel artificial membrane permeability (PAMPA) assay**

The PAMPA assay was performed by diluting the test compound in donor buffer (pH 7.4) to a concentration of 50 μM. Subsequently, 200 μL of this diluted test compound was added to the lower bottom of a 96-well transwell plate. The transwell plate's side facing the donor compartment was coated with a GIT lipid solution, and 150 μL of acceptor buffer (pH 7.4) was added to the upper part of the transwell plate. The assay was then incubated for 4 hours at 25 °C to allow for permeation across the lipid membrane. After the incubation period, samples from both the donor and acceptor compartments were collected and their UV absorbance was measured. The permeability rate was analyzed using the PAMPA explore program

**CYP4A enzyme activity assay**

HEK293T cells were cultured at 37 °C and in 5% CO_2_ in Dulbecco’s modified Eagle’s medium (DMEM; Thermo Fisher Scientific Inc., MA, USA) supplemented with 10% fetal bovine serum (Thermo Fisher Scientific Inc., MA, USA) and antibiotics. Transfection with CYP4A was performed using Lipofectamine 3000 (Invitrogen, CA, USA), and the cells were examined 16–24 h later. The cells were washed twice with cold PBS and extracted at 4 °C for 1 h in RIPA lysis buffer (Thermo Fisher Scientific Inc., MA, USA). The activities of HET0016 and CYP4A inhibitor candidates were compared using a Cytochrome P450 Enzyme Assay kit (Promega, WI, USA). CYP4A activity was measured using a P450-Glo assay kit with Luciferin-4A (Promega, WI, USA), following the manufacturer’s instructions. CYP4A activity was measured using a SpectroMax M4 (Molecular Devices, CA, USA). For the liver organoids, luciferase activity was quantified using a Victor X Light luminometer (PerkinElmer, MA, USA) and was normalized to the ATP content of each sample, measured using a CellTiter-Glo 3D cell viability assay kit (Promega, WI, USA).

**Cell culture and chemical treatments**

HepG2 cells, which were purchased from a Korean cell line bank (KCLB, Seoul, Korea), were cultured at 37 °C in 5% CO_2_ in DMEM (Thermo Fisher Scientific Inc., MA, USA) supplemented with 10% fetal bovine serum (Thermo Fisher Scientific Inc., MA, USA) and antibiotics. HepG2 cells (4 × 10^3^ cells per well for PA treatment, 1 × 10^4^ cells per well for thapsigargin (TH) or tunicamycin (TU) treatment) were seeded in black, clear-bottomed, 96-well culture plates (Corning, NY, USA). After overnight incubation, the cells were treated with 0.25 mM PA for 72 h, or thapsigargin (1 μM) or tunicamycin (5 μg/ml) for 24 h, then incubated with vehicle, HET0016 or CYP4A inhibitor candidates.

**Generation of 3D-based models of liver steatosis.**

For a 3D organoid-based model of liver steatosis, liver organoids were generated using a previously described protocol and maintained in a hepatic medium in a Matrigel™ (Corning, NY, USA) dome. After 3 days of division, 0.5 mM oleate (MilliporeSigma, MA, USA) and 0.25 mM palmitate (MilliporeSigma, MA, USA) with 12% fatty acid-free BSA (MilliporeSigma, MA, USA) were added to induce steatosis, along with 5 µM of each CYP4A inhibitor, and the cells were incubated for 3 days. For a 3D HepaRG-based model of liver steatosis, liver spheroids were formed as previously described from HepaRG cells (HPR101, Biopredic International, Saint-Gregoire, France), human umbilical vein endothelial cells, and human umbilical cord blood mesenchymal stem cells. These cells were seeded at an 8:1:1 ratio onto a presolidified Matrigel bed; after 7 days of self-organization, 50 mM glucose (Thermo Fisher Scientific Inc., MA, USA) and 125 μM palmitate were added to induce steatosis, along with 5 µM each CYP4A inhibitor, and the cells were incubated for 5 days.

**Measurements of glucose uptake and consumption**

Glucose uptake was measured using a Glucose Uptake Cell-Based Assay Kit (Cayman, MI, USA), following the manufacturer’s protocol. For HepG2 cells or 3D liver steatosis models, 100 or 200 μg/ml of 2-NBDG, a fluorescent glucose analog, were added in a glucose-free buffer for 30 min (HepG2 cells) or 4 h (3D liver steatosis models). For the HepG2 cells, glucose uptake was measured using a SpectroMax M4 (Molecular Devices, CA, USA); for 3D liver steatosis models, fluorescence imaging was performed using a Zeiss confocal microscope, and the normalized fluorescence intensity (RFI) was quantified using the ZEN program (Zeiss, Oberkochen, Germany). Glucose consumption was analyzed using a Glucose Assay Kit (MilliporeSigma, MA, USA). The glucose content of the culture medium of the organoids after 48 h of culture was subtracted from the content in the fresh medium.

**Staining for lipid droplets**

For *in vitro* models, cells were fixed with 4% paraformaldehyde solution (Thermo Fisher Scientific Inc., MA, USA) for 15 min at room temperature, then washed twice with PBS and incubated with Nile Red solution (1 μg/ml) at 37 °C for 10 min in the dark. Representative images were obtained using an Opera QEHS microscope with a 20× water lens, and the RFI was quantified using Columbus software (PerkinElmer, MA, USA). For Oil red O and Nile red staining in 3D liver steatosis models, organoids or frozen sections of spheroids were fixed in 4% paraformaldehyde (PFA, MilliporeSigma, MA, USA) at 4 °C overnight, then washed with 60% isopropanol (MilliporeSigma, MA, USA) for 5 min and incubated with Oil red O solution (MilliporeSigma, MA, USA) for 30 min at room temperature. The samples were then washed with distilled water, and images of the lipid were captured using an Olympus microscope. For quantification, the Oil red O stain was eluted using 100% isopropanol and absorbances were measured using a SpectroMax M3 microplate reader (Molecular Devices, CA, USA) and normalized to cell viability determined using a Cell Counting Kit 8 (Abcam, Cambridge, UK). Additionally, fixed organoids or frozen sections of spheroids were incubated with 10 μg/ml Nile Red solution (Thermo Fisher Scientific Inc., MA, USA) for 5 min at room temperature in the dark. Fluorescent images of the lipid were then obtained using confocal microscopy, and the RFI was quantified using the ZEN program.

**Measurement of ROS concentration**

ROS production was measured using cell-permeant 2′,7′-dichlorodihydrofluorescein diacetate (H2DCFDA; Thermo Fisher Scientific Inc., MA, USA). The cell culture medium was replaced by PBS containing 5 μM H2DCFDA. Representative images were obtained using an Opera QEHS microscope with a 20× water lens, and the RFI was quantified using Columbus software (PerkinElmer, MA, USA).

**Immunocytochemistry**

Liver spheroids were fixed in 4% PFA for 15 min at room temperature, incubated in 30% sucrose (MilliporeSigma, MA, USA), and then embedded in OCT compound (Sakura Finetek USA Inc., CA, USA). Frozen blocks were sectioned using a cryostat (Leica, Hesse, Germany), and the sections were permeabilized using 0.1% Triton X-100 (MilliporeSigma, MA, USA). The sections were then blocked with 4% BSA, incubated with specific primary antibodies at 4 °C overnight, and washed with 0.05% Tween-20 in PBS. Finally, they were incubated with the appropriate Alexa Fluor-conjugated secondary antibodies (Thermo Fisher Scientific Inc., MA, USA) for 1 h at room temperature, and fluorescence images were obtained using an Olympus microscope.

**Animal experiments**

For the T2DM animal model, CYP4A inhibitors (various concentrations; 1, 3, 5 mg/kg/day) or an equal volume of a vehicle were injected intraperitoneally into mice. For HFD-induced diabetes, 8-week-old male C57BL/6N mice were fed a normal chow diet (NCD) or high-fat diet (HFD) (60% of energy derived from fat, Research Diets Inc, NJ, USA) for 12 weeks. For genetic diabetes models, C57BL/KsJ-*db/db* mice were used as a genetic model of T2DM. Male C57BL/6N and C57BL/KsJ-*db/db* mice were injected intraperitoneally with CYP4A inhibitors (5 mg/kg/day) for 2 weeks while consuming the NCD. The body mass of each mouse and food intake were recorded every other day throughout the treatment period and at the time of sacrifice. For the MASH model, each CYP4A inhibitor (5 mg/kg/day) was injected intraperitoneally for 4 weeks into 6-week-old male *ob/ob* mice that were consuming an MCD diet (#A02082002B; Research diets Inc, NJ, USA) or an NCD. The body mass of each mouse was recorded every other day throughout the treatment period and at the time of sacrifice. All *in vivo* experiments were performed with at least three independent samples^2-5^.

**Glucose tolerance testing (GTT) and insulin tolerance testing (ITT)**

At the end of treatment, GTT and ITT were performed in mice fasted for 6 h following the intraperitoneal injection of 1 g/kg glucose or 0.75 U/kg insulin (Humulin N, Eli Lilly and Company, IN, USA) dissolved in PBS, respectively. Tail-blood glucose concentrations were determined before (0 min) and 15, 30, 60, 90, and 120 min after glucose or insulin injection.

**Hematoxylin and eosin (H&E) staining**

Liver samples were fixed in neutral-buffered formalin solution (MilliporeSigma, MA, USA-Aldrich), embedded in paraffin, and sectioned at 5 μm thickness. Briefly, the sections were dewaxed, hydrated, and stained with hematoxylin and eosin, then images of random fields were obtained using a light microscope (Leica, Hesse, Germany).

**Sirius red staining**

Liver samples were embedded in paraffin, sectioned, and stained with Picrosirius Red (MilliporeSigma, MA, USA-Aldrich), which stains collagen fibers. Images of random fields were obtained using a light microscope (Leica, Hesse, Germany).

**Measurement of triglyceride concentration**

Organoids or spheroids were homogenized in 5% NP-40 solution (MilliporeSigma, MA, USA) and heated at 80–100 °C for 5 min. To fully solubilize the triglyceride, the samples were cooled to room temperature, and then the heating and cooling steps were repeated. The samples were centrifuged, and the supernatants were diluted 10-fold with distilled water. Triglyceride concentration was measured using a Triglyceride Assay Kit (Abcam, Cambridge, UK) and a microplate reader, according to the manufacturer’s instructions. For mouse livers, triglyceride concentration was measured using a Triglyceride Colorimetric Assay kit (Cayman, MI, USA), following the manufacturer’s protocol. Briefly, mouse livers were homogenized in NP-substitute assay reagent, and the supernatants were diluted at least 1:5. The final sample concentrations were calculated using the absorbances at 530–550 nm and a standard curve.

**Lipid peroxidation assay**

The intracellular MDA concentration was measured in cell or mouse liver lysates using a Lipid Peroxidation Assay Kit (MilliporeSigma, MA, USA), according to the manufacturer’s instructions. MDA is an end-product of lipid peroxidation, which occurs as a result of oxidative damage and is a marker of oxidative stress.

**20-Hydroxyeicosatetraenoic acid (20-HETE) production**

The 20-HETE concentration was measured in mouse liver lysates using a 20-HETE ELISA Kit (Detroit R&D Inc., MI, USA)^6-8^, according to the manufacturer’s protocol. To perform the assay, all samples are loaded with blank, maximum binding wells, and six-point standards. The final sample concentrations were calculated using the absorbances at 450 nm and a standard curve.

**Serum chemistry**

Basal blood glucose concentration was measured using an automatic glucose monitor (One Touch, Lifescan, CA, USA). Plasma triglyceride, cholesterol, high-density lipoprotein (HDL), low-density lipoprotein (LDL), and free fatty acid concentrations were measured using colorimetric assay kits (Wako Pure Chemical Industries Ltd., Osaka, Japan). Insulin was measured using a Mouse Insulin ELISA kit (ALPCO, NH, USA). The activities of alanine aminotransferase (ALT) and aspartate transaminase (AST) were measured using an Activity Assay Kit (MilliporeSigma, MA, USA-Aldrich).

**Western blot analysis**

Cells or mouse liver tissue were washed twice with cold PBS and lysed at 4 °C for 1 h in RIPA lysis buffer (Thermo Fisher Scientific Inc., MA, USA), and their protein concentrations were determined using a BCA assay kit (Thermo Fisher Scientific Inc., MA, USA). Cell lysates were separated by SDS-PAGE and transferred onto PVDF membranes (Bio-Rad, CA, USA). The membranes were blocked with blocking solution (5% BSA/TBST) for 1 h at room temperature and then incubated with specific primary antibodies for 16 h at 4 °C. After washing three times with TBST buffer, the membranes were incubated with HRP-conjugated secondary antibody (Thermo Fisher Scientific Inc., MA, USA), and specific bands were visualized using a luminescent image analyzer (ImageQuant LAS-4000 mini; GE Healthcare, IL, USA). Organoids or spheroids were lysed using RIPA buffer (Thermo Fisher Scientific Inc., MA, USA) containing a protease inhibitor cocktail (MilliporeSigma, MA, USA), and protein concentrations were determined using a Bradford Protein Assay Kit (Bio-Rad, CA, USA). Lysate proteins were separated by electrophoresis using Mini-PROTEAN TGX Gels (Bio-Rad, CA, USA) and transferred to PVDF membranes (Bio-Rad, CA, USA) using a Trans-Blot Turbo Transfer System (Bio-Rad, CA, USA). Membranes blocked with 5% skim milk (BD Biosciences, NJ, USA) were incubated with specific primary antibodies at 4 °C overnight and washed with 0.1% Tween-20 (MilliporeSigma, MA, USA) in PBS (Thermo Fisher Scientific Inc., MA, USA). The membranes were then incubated with the appropriate HRP-conjugated secondary antibodies (Santa Cruz Biotechnology, TX, USA) for 1 hour at room temperature. Signals were detected using Supersignal West Femto Chemiluminescent Substrate (Thermo Fisher Scientific Inc., MA, USA) and LAS-3000 (Fujifilm, Tokyo, Japan) and analyzed using Image Gauge software (Fujifilm, Tokyo, Japan).

**Real-time polymerase chain reaction**

RNA of cell lysates or mouse live tissues was isolated using Tri Reagent (Molecular Research Center Inc., OH, USA), and reverse transcription was performed using a SuperScript III Reverse Transcriptase (Thermo Fisher Scientific Inc. (Invitrogen), MA, USA), according to the manufacturer’s instructions. Quantitative real-time PCR was performed using SYBR green master mix (Thermo Fisher Scientific Inc. (Applied Biosystems), MA, USA), and the results were analyzed using a QuantStudio3 Real-Time PCR System (Thermo Fisher Scientific Inc. (Applied Biosystems), MA, USA). For Organoids or spheroids, RNA was extracted from samples using Trizol (Thermo Fisher Scientific Inc., MA, USA), and reverse transcription was performed using a Topscript RT DryMIX (Enzynomics, Daejeon, Korea), according to the manufacturer’s instructions. Quantitative real-time PCR was performed using Fast SYBR Green Master Mix (Thermo Fisher Scientific Inc. (Applied Biosystems), MA, USA) and analyzed using a 7500 Fast Real-Time PCR System (Thermo Fisher Scientific Inc. (Applied Biosystems), MA, USA).

**Samples of liver from patients with NAFLD**

Liver samples from nontumor-affected regions were collected from six patients without steatosis, six patients with simple steatosis, and six patients with MASH who underwent hepatectomy at Severance Hospital because of liver cancer. The liver histology of the patients was classified by an experienced pathologist using published diagnostic criteria which included the presence of biopsy-confirmed steatosis, lobular inflammation, and/or fibrosis, without a history of substantial alcohol intake (< 210 g/week for men and 140 g/week for women) or hepatitis B/C virus infection. Informed consent was obtained from all participants, and the protocol was approved by the Institutional Review Board of Severance Hospital, Yonsei University College of Medicine (IRB No 4–2014–0674).

**Analysis of RNA expression in liver samples from patients**

RNA was isolated from human liver samples using TRIzol™ Reagent (Thermo Fisher Scientific Inc. (Invitrogen), MA, USA), according to the manufacturer’s instructions. cDNA was synthesized from 2 μg of RNA using Moloney Murine Leukemia Virus reverse transcriptase (Promega, WI, USA) and oligo(T) primer at 42 °C for 1 h. An aliquot (1/80 vol) of the cDNA was then subjected to PCR amplification using human gene-specific primers. Real-time RT-PCR was conducted using SYBR Green Master Mix (Takara, CA, USA) in a QuantStudio 3 Real-Time PCR machine (Thermo Fisher Scientific Inc. (Applied Biosystems), MA, USA). The normalized expression of CYP4A11 was normalized to that of L32 mRNA.

**RNA-Seq Analysis**

Total RNA was extracted from mouse liver tissues using Tri Reagent (Molecular Research Center Inc., OH, USA), according to the manufacturer’s instructions. RNA quality was evaluated by analyzing the RNA integrity number determined using a 2100 bioanalyzer (Agilent RNA 6000 Nano kit, Agilent Technologies, CA, USA). Library preparation was performed using Advanta RNA-seq XT NGS Library Prep kits for Fluidigm JUNO. Sequencing was conducted on an Illumina NextSeq 550. The raw FASTQ files were filtered by Trimmomatic. Filtered FASTQ files were mapped and counted with STAR 2.7. Significant genes were identified by using the cut-off values of *p*-value < 0.05 and fold change (FC) in the mean expression of |FC| > = 1.5. Volcano plots of differentially expressed genes (DEGs) were generated using R packages.

**Bioinformatic pathway analysis**

GSEA was performed to investigate the rank genes according to expression variation between the group of HFD and HFD treated with CYP4A inhibitors in the enrichment of the MSigDB Collection. For DEG network and pathway analysis, Ingenuity Pathway Analysis (IPA) was performed. The list of DEGs was uploaded into QIAGEN’s Ingenuity Pathway Analysis (IPA) to carry out core analysis. As a result, core analysis 1 (NCD + VE compared with HFD + VE) and core analysis 2 (HFD + VE compared with HFD + C1 or C2) were obtained. Then, Comparison Analysis were implemented to visualize the results across multiple analyses with varying conditions to identify trends or similarities and differences between conditions. Biological networks and pathways were analyzed to study the direct or predicted relationships.

**Statistical analysis**

Data are shown as the mean ± SEM. GraphPad Prism 5.0 software was used to perform two-way ANOVA or two-tailed unpaired Student’s *t*-tests to analyze the data. *P* < 0.05 was considered to indicate statistical significance. All results were obtained from at least three independent experiments.

**Table S1 % Inhibition of HET0016 or CYP4A Inhibitors and Metabolic stability**

|  | | **CYP inhibition assay (IC_50_) ^a^** | | | | | | | | | **Microsomal stability**  **(% Remaining)** | |
| --- | --- | --- | --- | --- | --- | --- | --- | --- | --- | --- | --- | --- |
|  |  |  |  |  |  |  |  |  |  |  | **Human** | **Rat** |
|  | **CYP1A2** | | **CYP2C9** | | **CYP2C19** | | **CYP2D6** | | **CYP3A4** | | **30 min** | |
| **HET0016** | | 0.2* | | 6.3** | | 1.3** | | 10.2*** | | >50*** | 13.7 | 4.2 |
| **C1** | | >100*** | | 23.4*** | | 10.7*** | | 12.7*** | | 6.8** | 40.9 | 40.0 |
| **C2** | | >100*** | | 41.4*** | | 53.1*** | | 20.8*** | | 30.0*** | 62.3 | 65.3 |
| **Ketoconazole**  **(% of control)** | | >100*** | | >100** | | >100** | | >100** | | 24.7 |  | |

^a^ Potent inhibition: *IC_50_ < 1 μM, **Moderate inhibition:1 μM < IC_50_ < 10 μM, ***No or Weak inhibition: IC_50_ > 10 μM; The potent inhibition of drugs on major CYPs related to hepatotoxicity and their microsomal stability. Through the CYP inhibition assay, C1 moderately inhibited CYP3A4 activity, while exhibiting no or weak inhibitory effects on other tested CYPs. Similarly, C2 displayed negligible inhibitory effects on five CYPs, suggesting minimal potential for clinical interactions. Furthermore, CYP4A inhibitors significantly enhanced metabolic stability in both rat and human liver microsomes compared to HET0016.

Table S2 % Plasma stability and permeability

| **Compound** | **Plasma Stability**  **(% Remaining)** | | | | **PAMPA ^a^** | | |
| --- | --- | --- | --- | --- | --- | --- | --- |
|  | **Human** | | **Rat** | | **GI** | | |
|  | **30min** | **120 min** | **30min** | **120 min** | **P_e_**  **(10^-6^ cm/sec)** | **BCS code** | **Solubility** |
| **C1** | > 100 | > 100 | > 100 | 98.2 | 90.23 | High | High |
| **C2** | > 100 | > 100 | > 100 | 98.2 | 93.604 | High | High |

^a^ Permeability classification, P_e_ (10-6 cm/sec): High > 1.5, Low < 1.5

**Table S3 Physiological and Serum Chemistry Results from HFD Mice.**

All experiments were performed as multiple independent samples, and the values are shown as mean ± SEM, analyzed by the two-way ANOVA. *n*=3-4; **P* < 0.05, ***P* < 0.01, ****P* < 0.001 for NCD + VE vs HFD + VE vs HFD + C1 or C2.

|  | **NCD + VE** | **HFD + VE** | **HFD + C1** | **HFD + C2** |
| --- | --- | --- | --- | --- |
| **Body mass (g)** | 30.89 ± 0.91 | 47.65 ± 1.14 | 37.04 ± 1.33*** | 36.9 ± 0.83*** |
| **Fat pad mass (g)** | 0.63 ± 0.08 | 1.55 ± 0.03 | 1.28 ± 0.04*** | 1.37 ± 0.009*** |
| **Liver mass (g)** | 1.25 ± 0.07 | 1.89 ± 0.12 | 1.06 ± 0.12** | 1.09 ± 0.04** |
| **Glucose (mmol/L)** | 12.79 ± 0.9 | 16.48 ± 0.62 | 12.01 ± 1.22* | 11.54 ± 0.65** |
| **Insulin (mg/dL)** | 35.43 ± 2.14 | 191.3 ± 36.84 | 45.70 ± 7.75** | 61.07 ± 18.06** |
| **ALT (U/L)** | 26.03 ± 2.63 | 85.73 ± 10.29 | 41.9 ± 11.18* | 43.38 ± 2.92** |
| **AST (U/L)** | 43.1 ± 2.56 | 110.1 ± 2.5 | 67.95 ± 7.42*** | 77.78 ± 7.44*** |
| **TG (mg/dL)** | 48.06 ± 2.18 | 59.89 ± 3.09 | 39.25 ± 3.11* | 38.37 ± 0.3* |
| **Total cholesterol (mg/dL)** | 71.21 ± 5.41 | 154.9 ± 12.86 | 68.22 ± 2.24*** | 74.46 ± 2.41*** |
| **LDL cholesterol (mg/dL)** | 7.89 ± 0.13 | 29.08 ± 2.65 | 9.53 ± 0.32*** | 9.86 ± 0.44*** |
| **LDL/HDL ratio (mg/dL)** | 0.09 ± 0.004 | 0.22 ± 0.02 | 0.13 ± 0.01** | 0.11 ± 0.001*** |
| **Total adiponectin** | 29.00 ± 3.51 | 18.2 ± 1.4 | 28.07 ± 2.56* | 27.1 ± 0.15* |
| **HMW adiponectin** | 30.23 ± 3.41 | 18.87 ± 1.62 | 28.3 ± 0.86** | 26.47 ± 0.47* |

**Table S4 Comparison analysis of Ingenuity Canonical Pathways between HFD + C1 and HFD + C2.**

| **Ingenuity Canonical Pathways** | **-log(p-value)** | |
| --- | --- | --- |
|  | **HFD**  **vs**  **HFD + C1** | **HFD**  **vs**  **HFD + C2** |
| NRF2-mediated Oxidative Stress Response | 3.424 | 6.92 |
| Acute Phase Response Signaling | 6.003 | 2.709 |
| LPS/IL-1 Mediated Inhibition of RXR Function | 2.515 | 5.131 |
| Fcγ Receptor-mediated Phagocytosis in Macrophages and Monocytes | 4.836 | 1.868 |
| LXR/RXR Activation | 2.299 | 3.999 |
| Phagosome Formation | 3.302 | 2.737 |
| Aryl Hydrocarbon Receptor Signaling | 1.225 | 4.537 |
| FXR/RXR Activation | 2.262 | 3.2 |
| Atherosclerosis Signaling | 1.431 | 3.79 |
| Sirtuin Signaling Pathway | 0.652 | 4.42 |
| PXR/RXR Activation | 2.24 | 2.539 |
| Natural Killer Cell Signaling | 3.05 | 1.513 |
| STAT3 Pathway | 2.157 | 2.368 |
| IL-12 Signaling and Production in Macrophages | 2.146 | 2.351 |
| Oxidative Phosphorylation | 1.615 | 2.847 |
| Glutathione-mediated Detoxification | 0.728 | 3.668 |
| Glucocorticoid Receptor Signaling | 1.787 | 2.508 |
| Xenobiotic Metabolism CAR Signaling Pathway | 1.044 | 3.213 |
| Pregnenolone Biosynthesis | 2.005 | 2.128 |

**Table S5. Comparative Analysis of the Top 10 Down-Regulated DEGs by C1 and C2 Treatments in HFD-Fed Mice.**

| **HFD vs HFD + C1** | | | **HFD vs HFD + C2** | | |
| --- | --- | --- | --- | --- | --- |
| **Gene Symbol** | **Log2 FC** | ***P*-value** | **Gene Symbol** | **Log2 FC** | ***P*-value** |
| **Gm39584 *** | -2.884 | 1.E-04 | **CISH *** | -3.595 | 6.E-26 |
| **CISH *** | -2.835 | 1.E-02 | **CIDEA *** | -3.136 | 2.E-04 |
| **Gm39447 *** | -2.377 | 3.E-06 | **Gm39584 *** | -2.685 | 3.E-04 |
| **GADD45G *** | -2.334 | 6.E-19 | SPRR1A | -2.529 | 1.E-04 |
| S100G | -2.316 | 5.E-03 | Egfros | -2.424 | 1.E-03 |
| Gm31522 | -2.278 | 2.E-02 | **GADD45G *** | -2.131 | 9.E-14 |
| SOCS2 | -2.207 | 3.E-07 | ID3 | -1.905 | 9.E-08 |
| **CIDEA *** | -1.983 | 6.E-24 | **Gm39447 *** | -1.895 | 8.E-04 |
| Gm7652 | -1.969 | 3.E-02 | PHLDA1 | -1.885 | 7.E-16 |
| SERPINE1 | -1.955 | 2.E-02 | TMEM200B | -1.801 | 3.E-02 |

The genes that are common between C1 and C2 treatments are indicated with bold text and an asterisk (*)


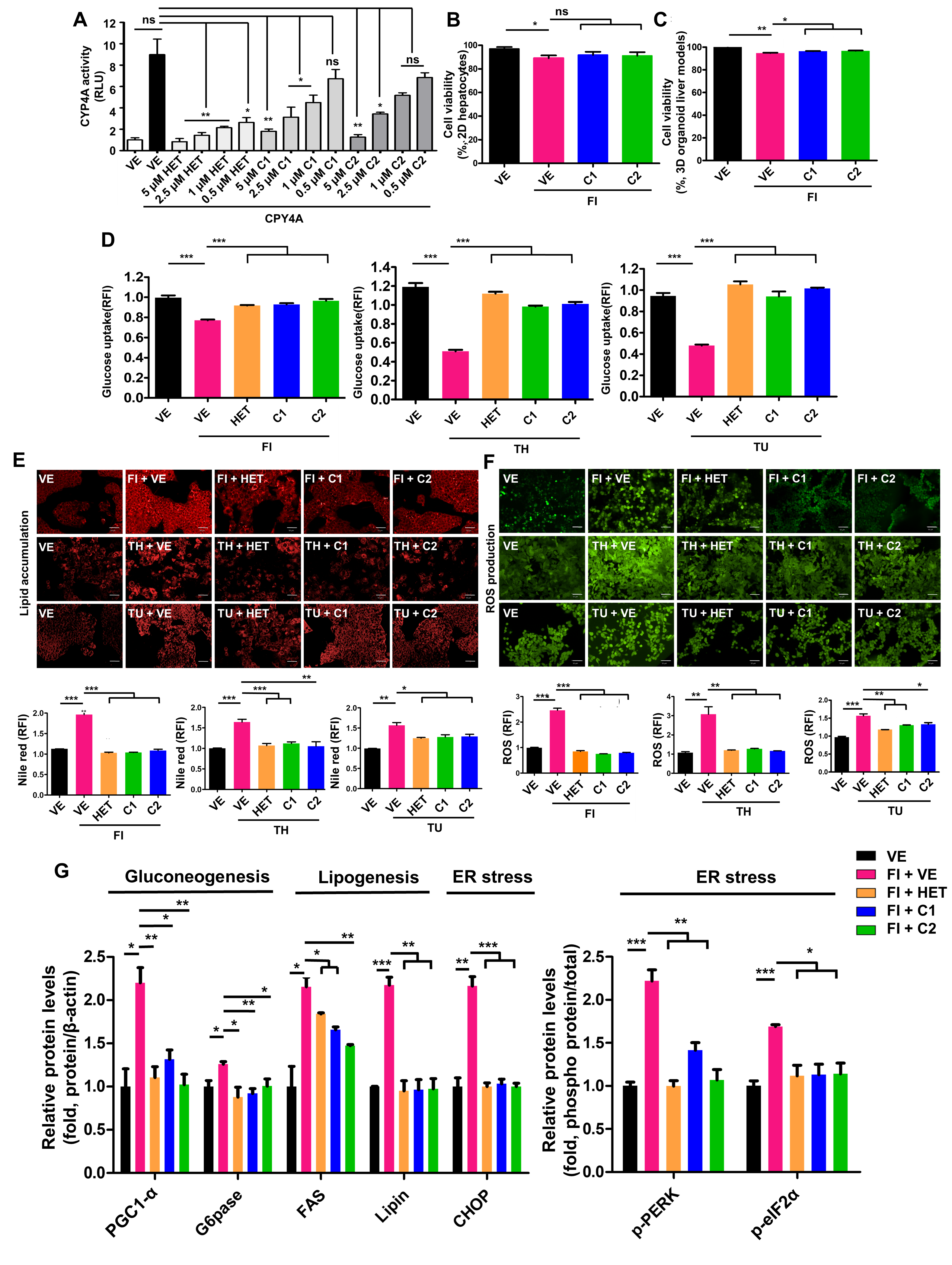


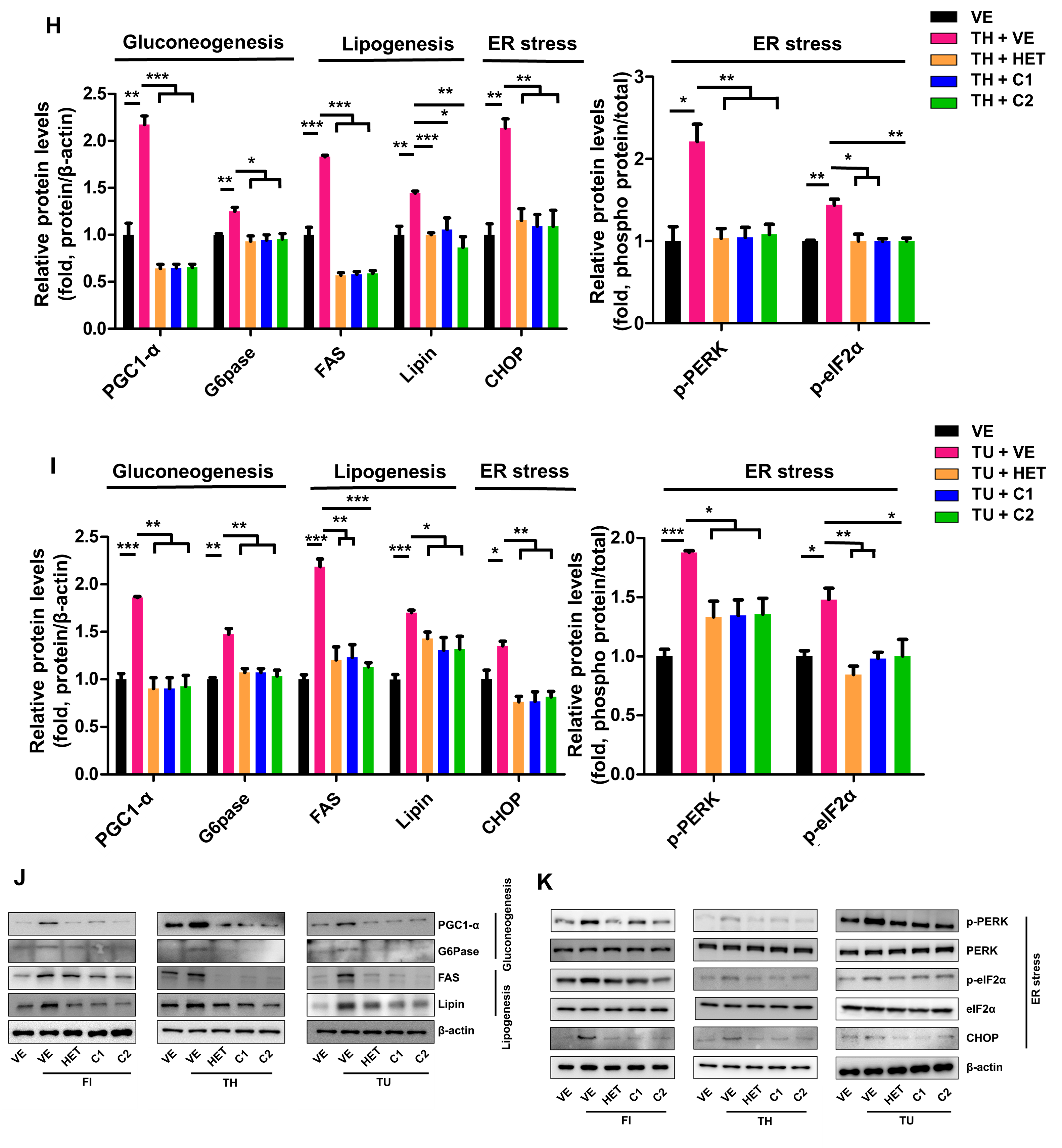


Figure S1 The effect of C418 derivatives, C1 and C2 *in vitro*. (A) Normalized CYP4A activity in the presence of HET0016 (HET), C1, or C2 at the concentration of 0.5, 1, 2.5, or 5 μM. (B-C) The effect of C1 or C2 on hepatic cytotoxicity assay in (B) *in vitro* or (C) 3D organoid liver steatosis models. (D-K) HepG2 cells were prepared by 0.25 mM palmitate (Fatty Induction, FI) or ER stress inducers (thapsigargin (TH) or tunicamycin (TU)) for the evaluation of CYP4A inhibitors (C1 and C2). (D) Representative fluorescence intensity (RFI) of glucose uptake. (E) Representative fluorescence images and intensity of Nile Red staining (scale bar = 50 μm). (F) Representative fluorescence images of ROS production (scale bar = 50 μm). (G-I) Quantification of western blot. Relative protein levels normalized to β-actin and phosphorylated protein levels normalized to total protein (G) Fatty Induction. (H) Thapsigargin. (I) Tunicamycin. (J) Western blots for proteins involved in gluconeogenesis and lipogenesis. (K) Western blots for mediators of ER stress. All experiments were performed as multiple independent samples, and the values are shown as mean ± SEM, analyzed by the Student’s *t*-test. *n*=3-5; **P* < 0.05, ***P* < 0.01, ****P* < 0.001 for VE vs FI or ER stress inducers + VE and FI or ER stress inducers + VE vs FI or ER stress inducers + HET, C1 or C2. ns, not significant.


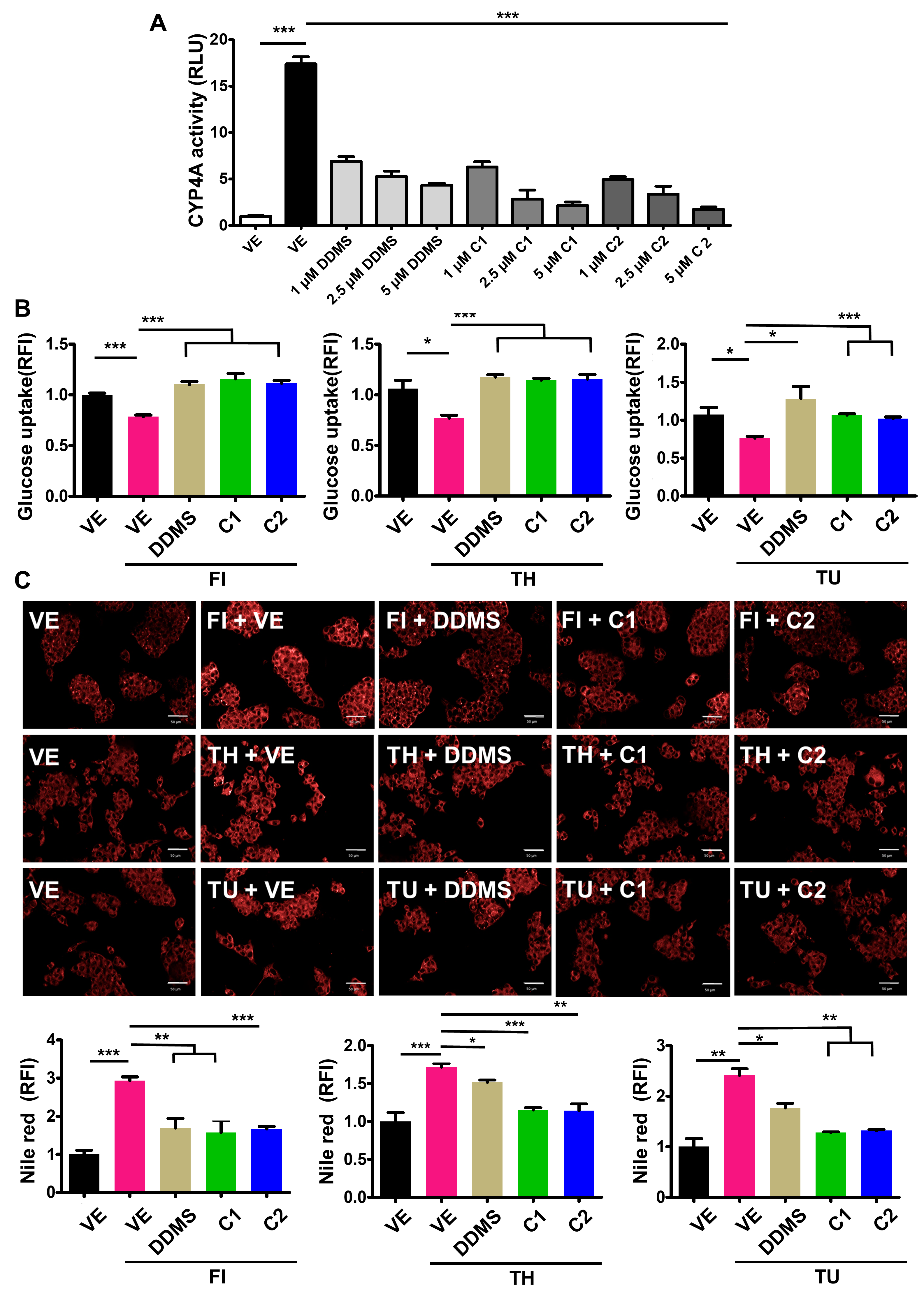


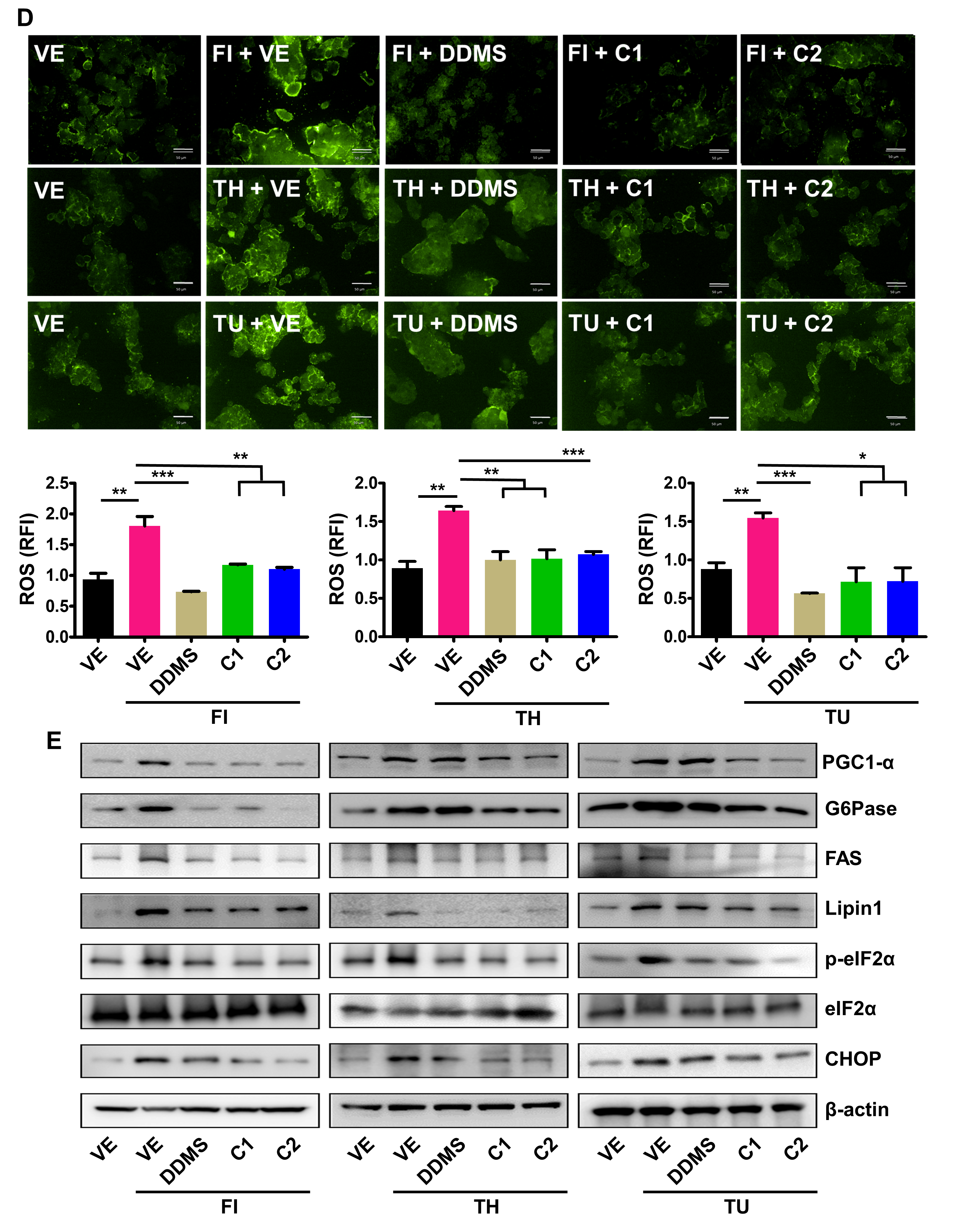


Figure S2 The effect of C1 and C2 *in vitro* compared to DDMS. The efficacy of C1 and C2 was evaluated in vitro compared to DDMS, a selective inhibitor of CYP4A. (A) Normalized CYP4A activity in the presence of DDMS, C1, or C2 at the concentration of 1, 2.5, or 5 μM. (B-D) HepG2 cells were prepared by 0.25 mM palmitate (Fatty Induction, FI) or ER stress inducers (thapsigargin (TH) or tunicamycin (TU)) for the evaluation of CYP4A inhibitors (C1 and C2). (B) Representative fluorescence intensity (RFI) of glucose uptake. (C) Representative fluorescence images and intensity of Nile Red staining (scale bar = 50 μm). (D) Representative fluorescence images of ROS production (scale bar = 50 μm). (E) Western blots of DDMS, C1, and C2. All experiments were performed as multiple independent samples, and the values are shown as mean ± SEM, analyzed by the Student’s *t*-test. *n*=3-5; **P* < 0.05, ***P* < 0.01, ****P* < 0.001 for VE vs FI or ER stress inducers + VE and FI or ER stress inducers + VE vs FI or ER stress inducers + DDMS, C1 or C2.


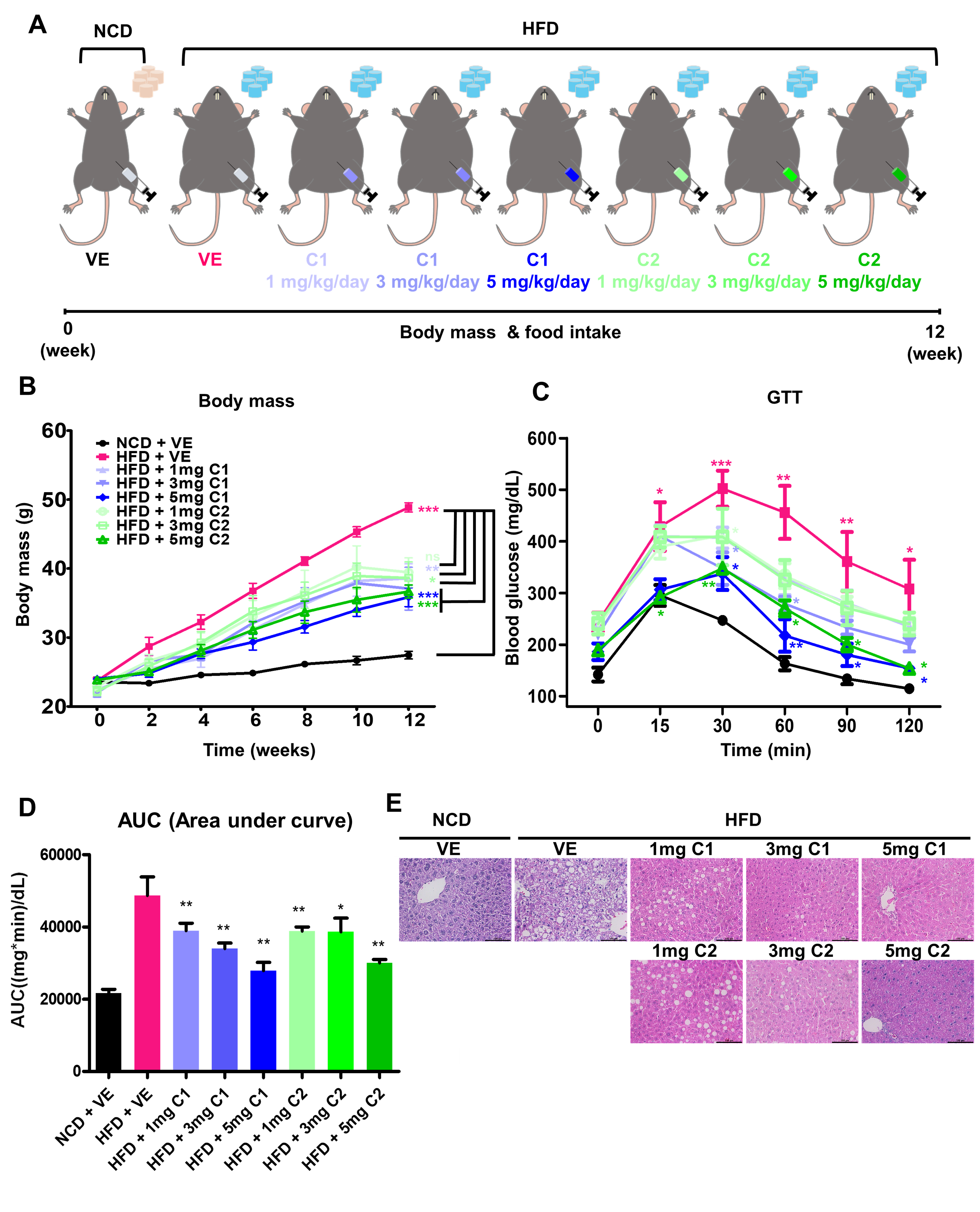


**Figure S3 Validation of C1 and C2 concentration in HFD-fed mice.** C57BL/6N mice were fed an NCD or HFD and administered with either vehicle (VE) or CYP4A inhibitors (C1 or C2, 1, 3, 5 mg/kg/day) intraperitoneally for 12 weeks. (A) Scheme of the C1 or C2 administration to HFD-fed mice. (B) Body masses of the mice. (C) GTT data. (D) AUC (Area under curve) of GTT data. (E) Representative H&E-stained liver sections (scale bar = 100 μm). All experiments were performed as multiple independent samples, and the values are shown as mean ± SEM, analyzed by two-way ANOVA. *n*=4; **P* < 0.05, ***P* < 0.01, ****P* < 0.001 for NCD + VE vs HFD + VE vs HFD + C1 or C2 (1, 3, 5 mg/kg/day)

**
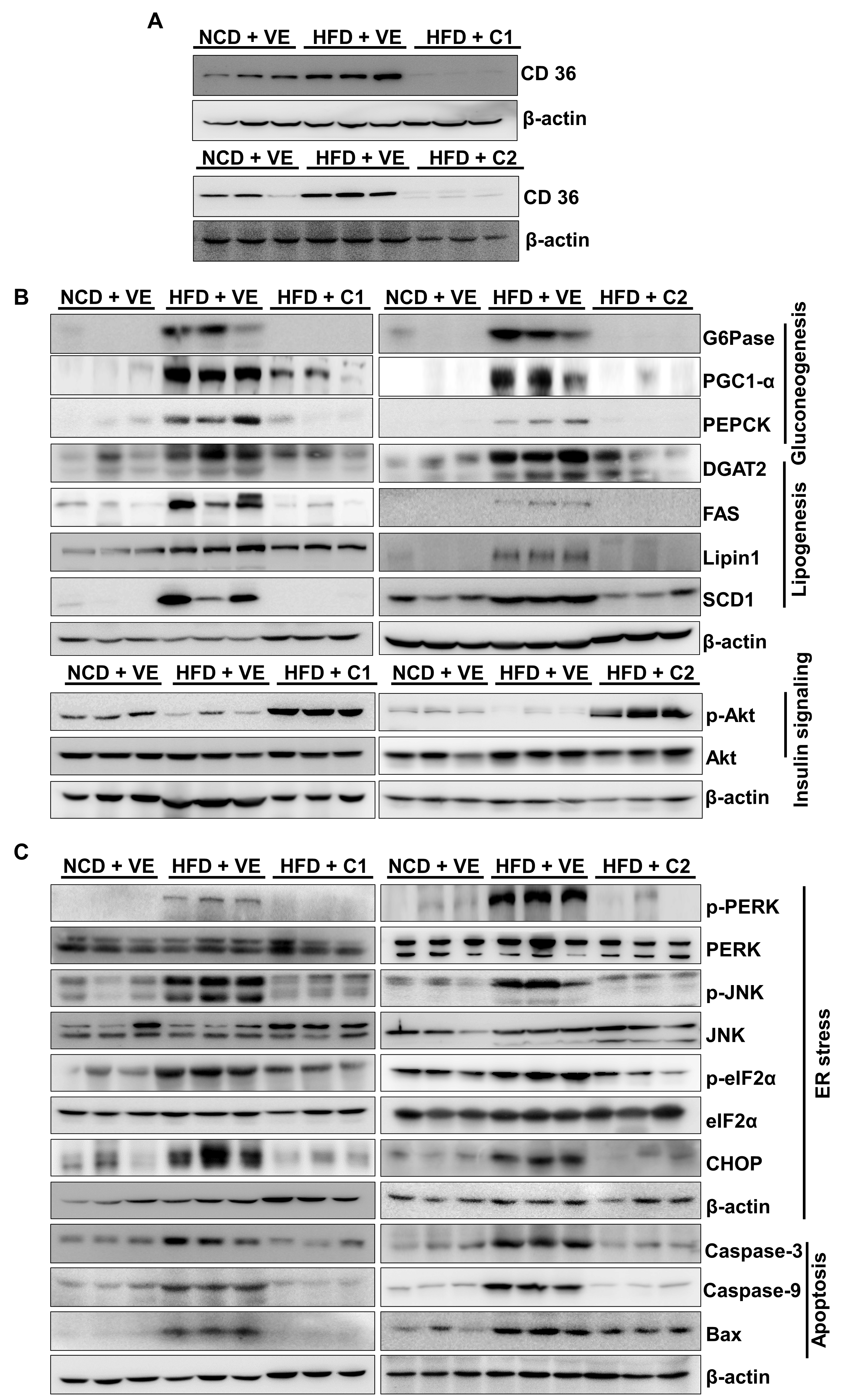
**

**Figure S4** **Effect of C1 and C2 in HFD-fed mice.** C57BL/6N mice were fed an NCD or HFD and administered with either vehicle (VE) or CYP4A inhibitors (C1 or C2, 5 mg/kg/day) intraperitoneally for 12 weeks. (A-C) Western blots of C1 and C2. (A) FAT/CD36. (B-C) Western blots of C1 and C2 on the gluconeogenesis, lipogenesis, ER stress, insulin resistance, and apoptosis of HFD-fed mice. All experiments were performed as multiple independent samples.


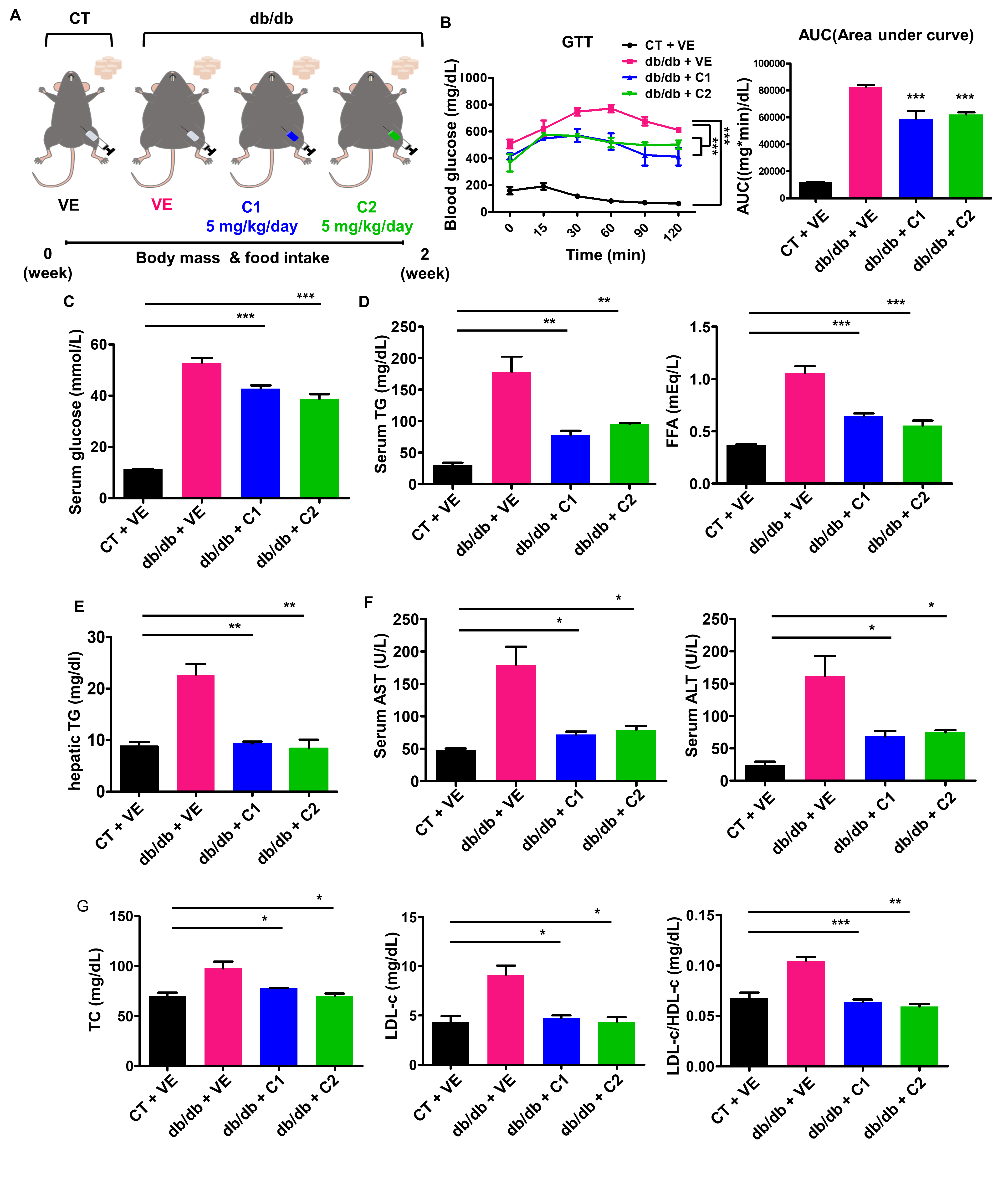

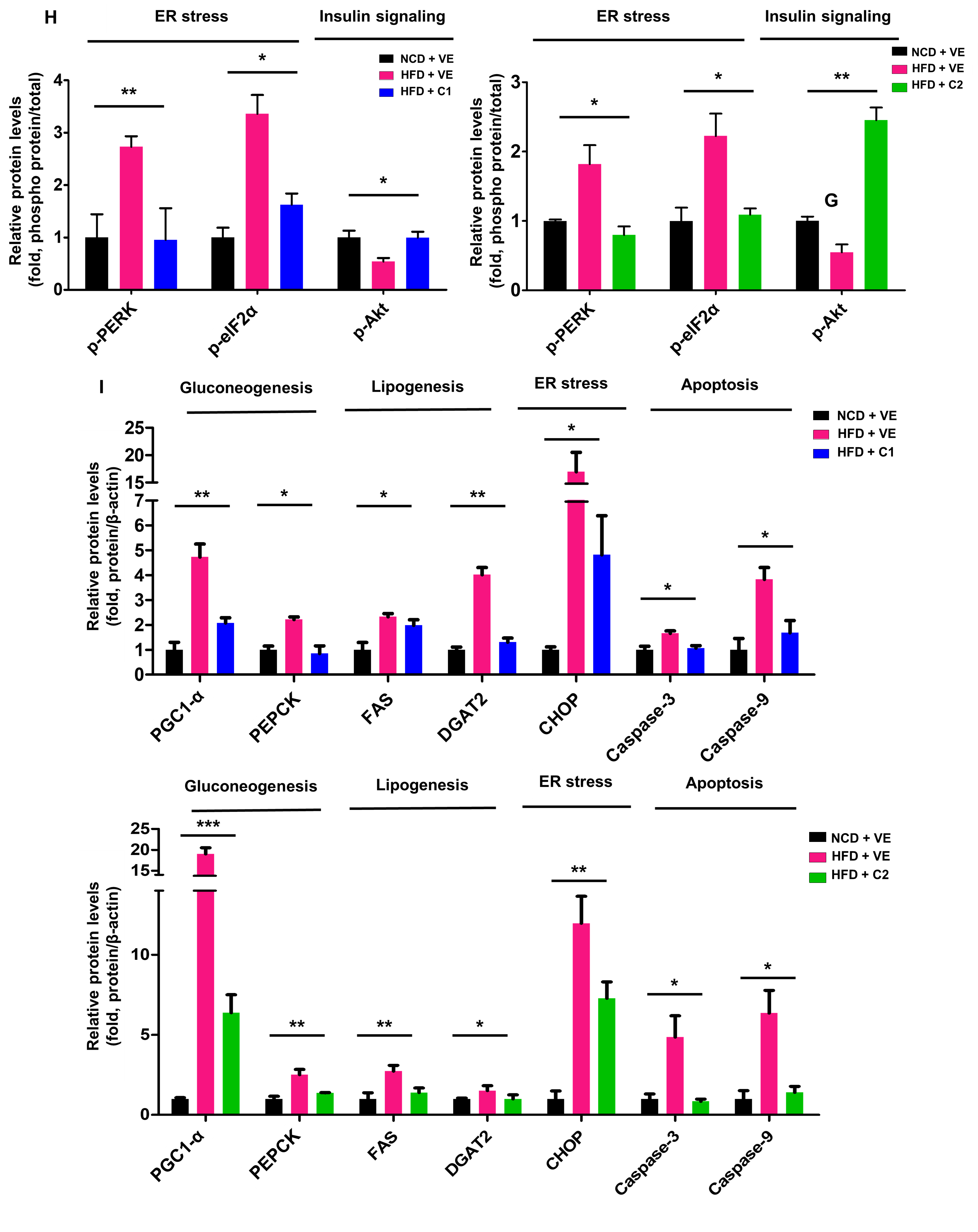


**
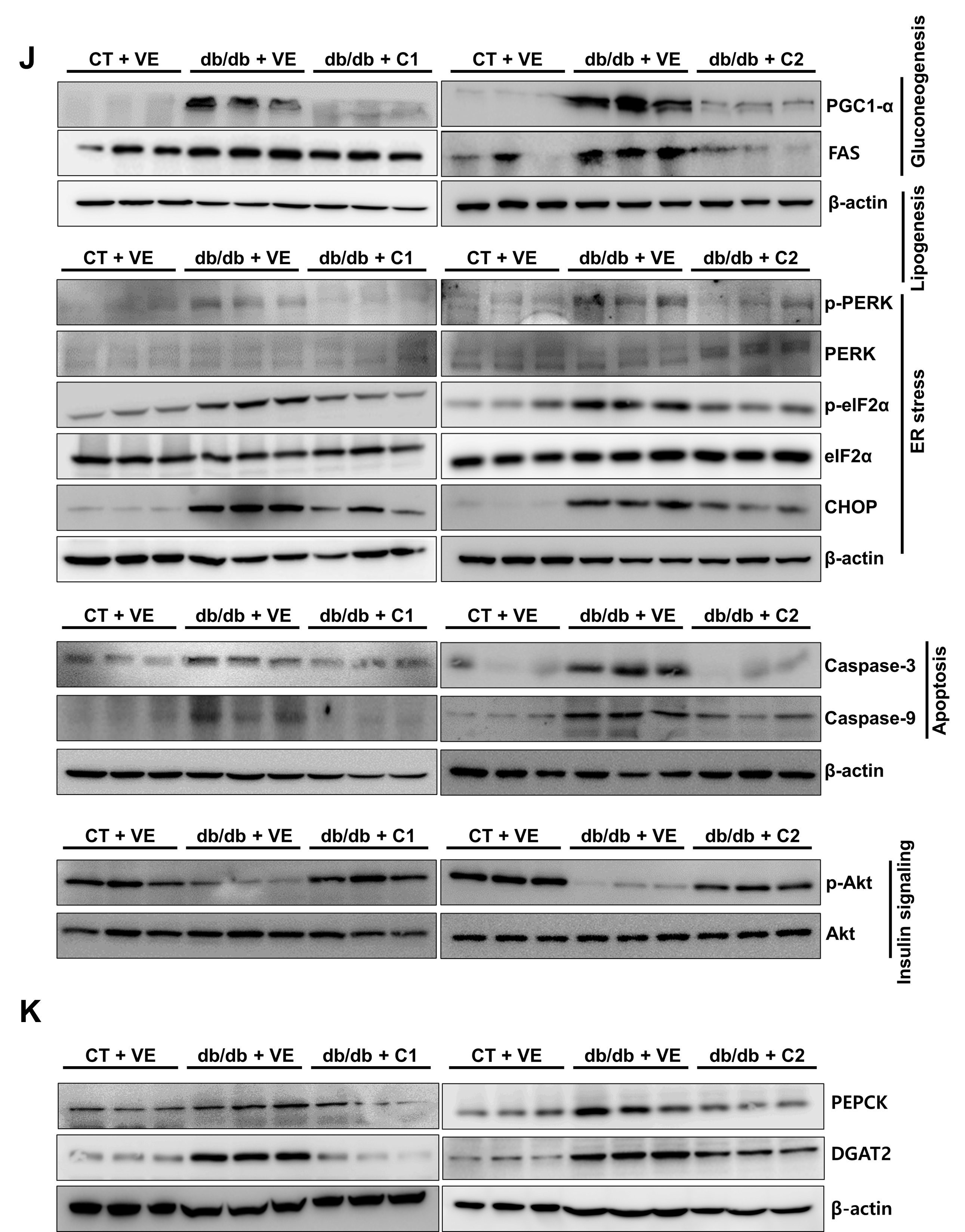
**

**Figure S5** **Effects of C1 and C2 in *db/db* diabetic mice.** C57BL/6N mice or diabetic mice (*db/db*) were administered either vehicle or CYP4A inhibitors (C1 or C2, 5 mg/kg/day) intraperitoneally for 2 weeks. (A) Scheme of the administration of C1 or C2 to diabetic mice. (B) GTT data and AUC (Area under curve) of GTT data. (C) Serum glucose levels. (D) Serum TG and free fatty acid (FFA) levels. (E) Hepatic TG concentration. (F) serum ALT and AST activities. (G) Serum lipid concentrations. (H-I) Quantification of western blot. (H) Normalization of Phosphorylated Protein Levels to Total Protein. (I) Relative protein levels normalized to β-actin expression. (J-K) Expression of proteins involved in gluconeogenesis, lipogenesis, ER stress, insulin signaling, and apoptosis in liver lysates. All experiments were performed as multiple independent samples, and the values are shown as mean ± SEM, analyzed by two-way ANOVA. *n*=3; **P* < 0.05, ***P* < 0.01, ****P* < 0.001 for NCD + VE vs *db/db* + VE vs *db/db* + C1 or C2.


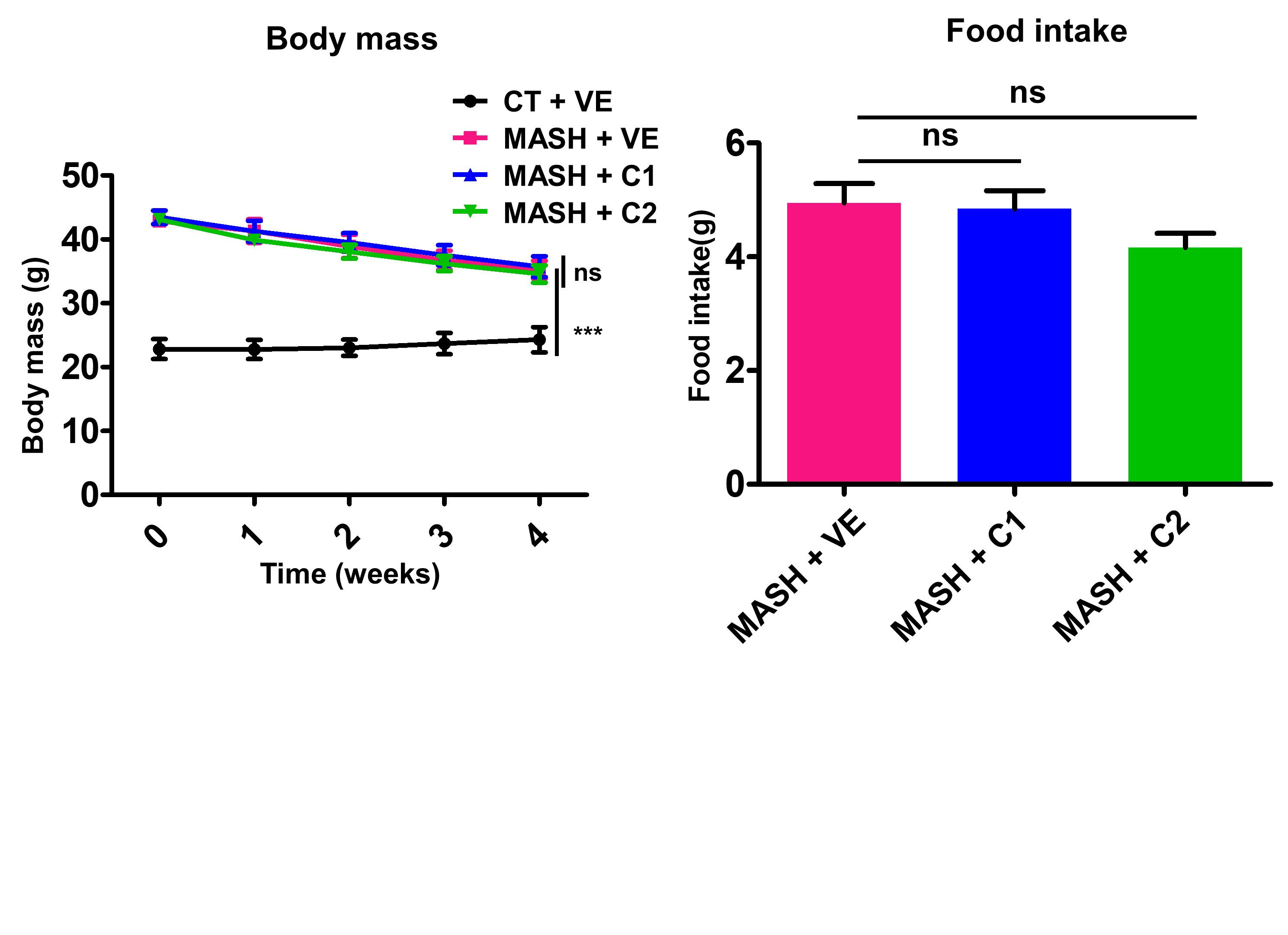


Figure S6 Body mass and food intake in MASH model. C57BL/6N and MASH model, respectively, and administered either vehicle (VE) or one of the candidate drugs (C1 or C2, 5 mg/kg/day) intraperitoneally for 4 weeks. (A) Body mass. (B) Food intake. All experiments were performed as multiple independent samples, and the values are shown as mean ± SEM, analyzed by the two-way ANOVA. *n*=3; ****P* < 0.001 for CT (CT; control mice) + VE vs MASH + VE vs MASH + C1 or C2. ns, not significant.


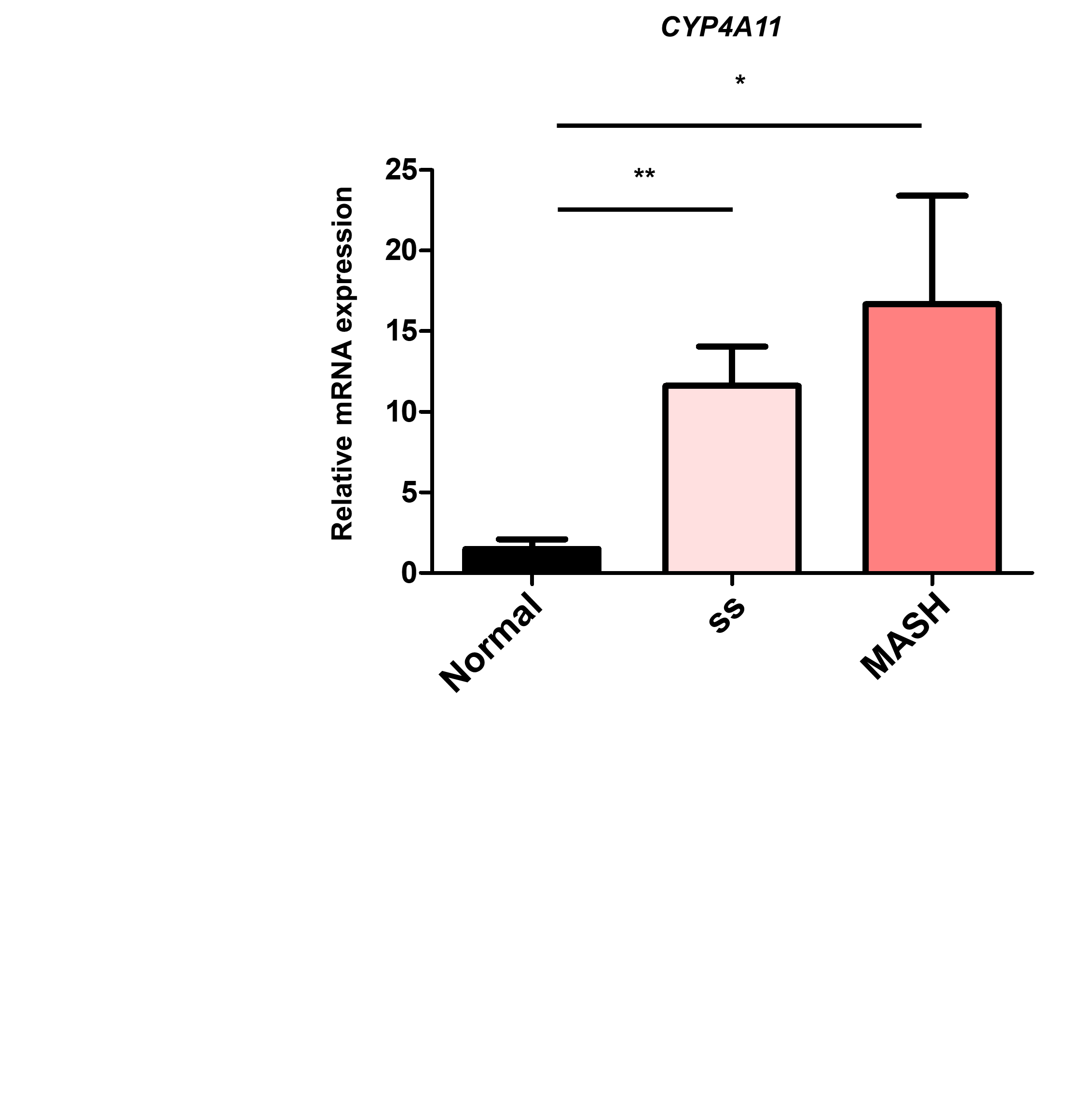


Figure S7 Normalized CYP4A11 mRNA expression in livers of patients with simple steatosis (SS) or Metabolic dysfunction-associated steatohepatitis (MASH) and controls. All experiments were performed as multiple independent samples, and the values are shown as mean ± SEM, analyzed by analyzed by the Student’s *t*-test. *n*=6; **P* < 0.05, **P < 0.01 for Normal vs SS and Normal vs MASH.


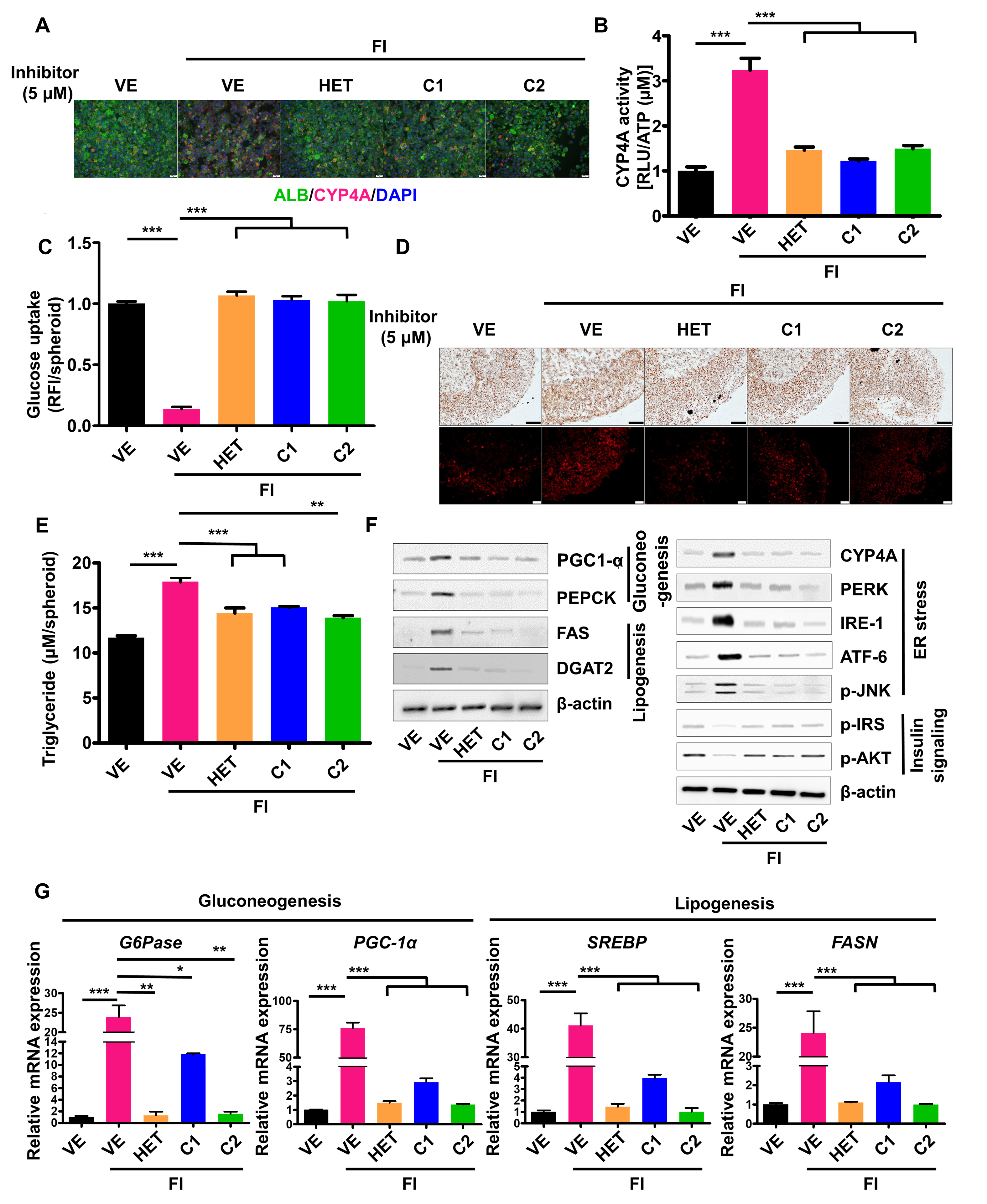


**Figure S8 Effects of C1 and C2 in a 3D HepaRG-based model of liver steatosis.** 3D HepaRG-based model of liver steatosis was generated with high 50 mM glucose and 125 μM palmitate (Fatty Induction, FI) for 5 days. For Fatty induction, hepatic fatty acid acts as endogenous ligands for peroxisome proliferator receptor alpha (PPARα), which is responsible for lipid metabolism and also plays a crucial role in glucose homeostasis. PPARα signaling increases the expressions of target genes, including those involved in gluconeogenesis (G6Pase and PEPCK), and improves insulin resistance. (A) Representative fluorescence image of ALB and CYP4A expression in a 3D HepaRG-based model of steatosis treated with vehicle (VE), HET0016 (HET), or CYP4A inhibitors (C1 or C2). (B) CYP4A enzyme activity. (C) Glucose uptake. (D) Intracellular lipid staining with Oil red O (*top*) and Nile Red (*bottom)*. (E) Triglyceride concentration. (F) Western blots for mediators of gluconeogenesis, lipogenesis, ER stress, and insulin sensitivity. (G) mRNA expression of genes involved in gluconeogenesis and lipogenesis. All experiments were performed as multiple independent samples, and the values are shown as mean ± SEM, analyzed by the Student’s *t*-test. *n*=3-5; **P* < 0.05, ***P* < 0.01, ****P* < 0.001 for VE vs FI + VE and FI + VE vs FI + HET or C1 or C2.


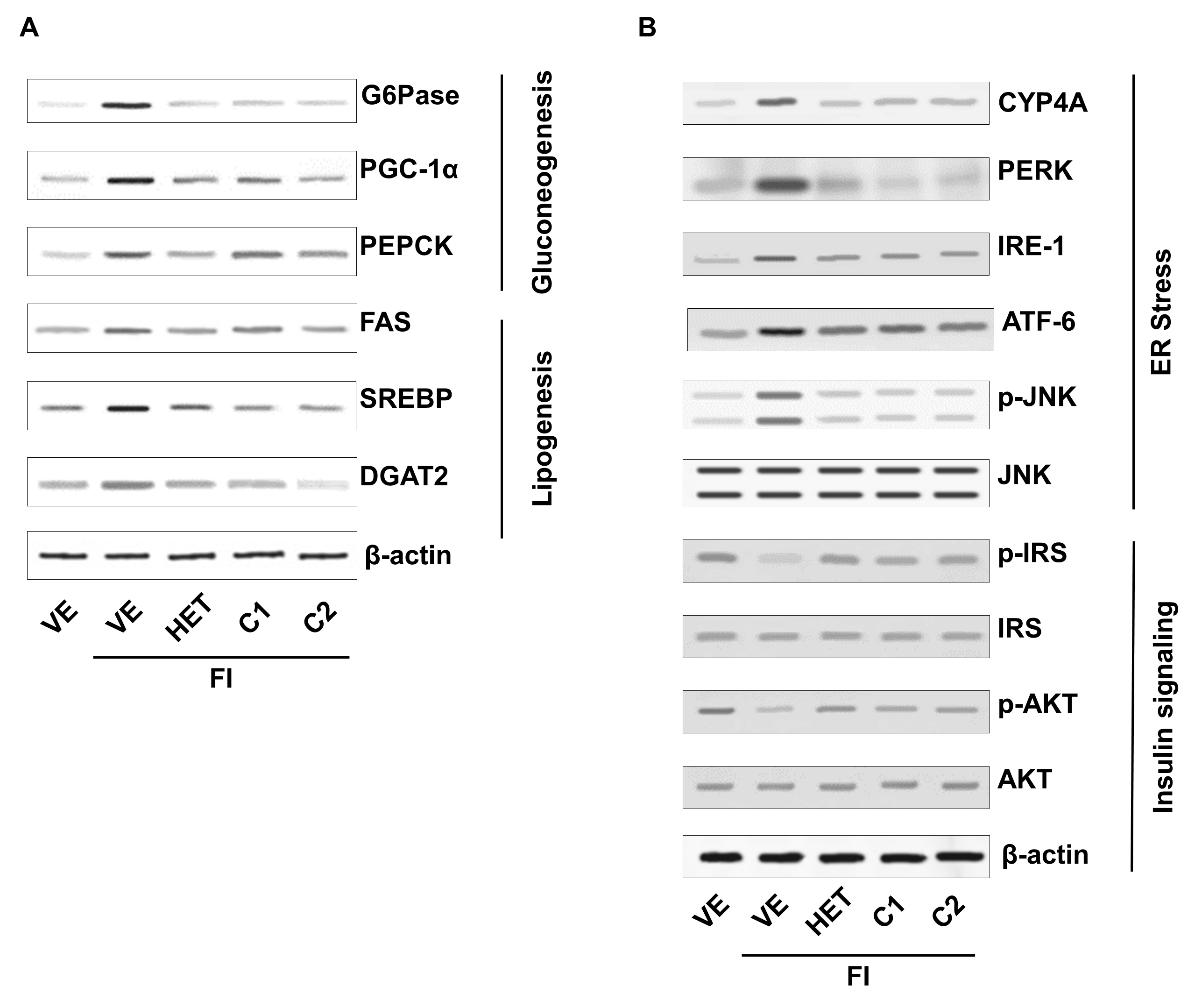


**Figure S9** **Effects of C1 and C2 in a 3D organoid-based model of liver steatosis.** Treatment with vehicle (VE), HET0016 (HET), or CYP4A inhibitors (C1 or C2) for 3 days in an organoid model of 0.5 mM oleate and 0.25 mM palmitate-induced liver steatosis (Fatty Induction, FI). (A-B) Western blots of C1 and C2. (A) Western blots for expression markers involved in gluconeogenesis and lipogenesis (B) assessment of ER stress and insulin signaling marker expression via western blot. All experiments were performed as multiple independent samples.


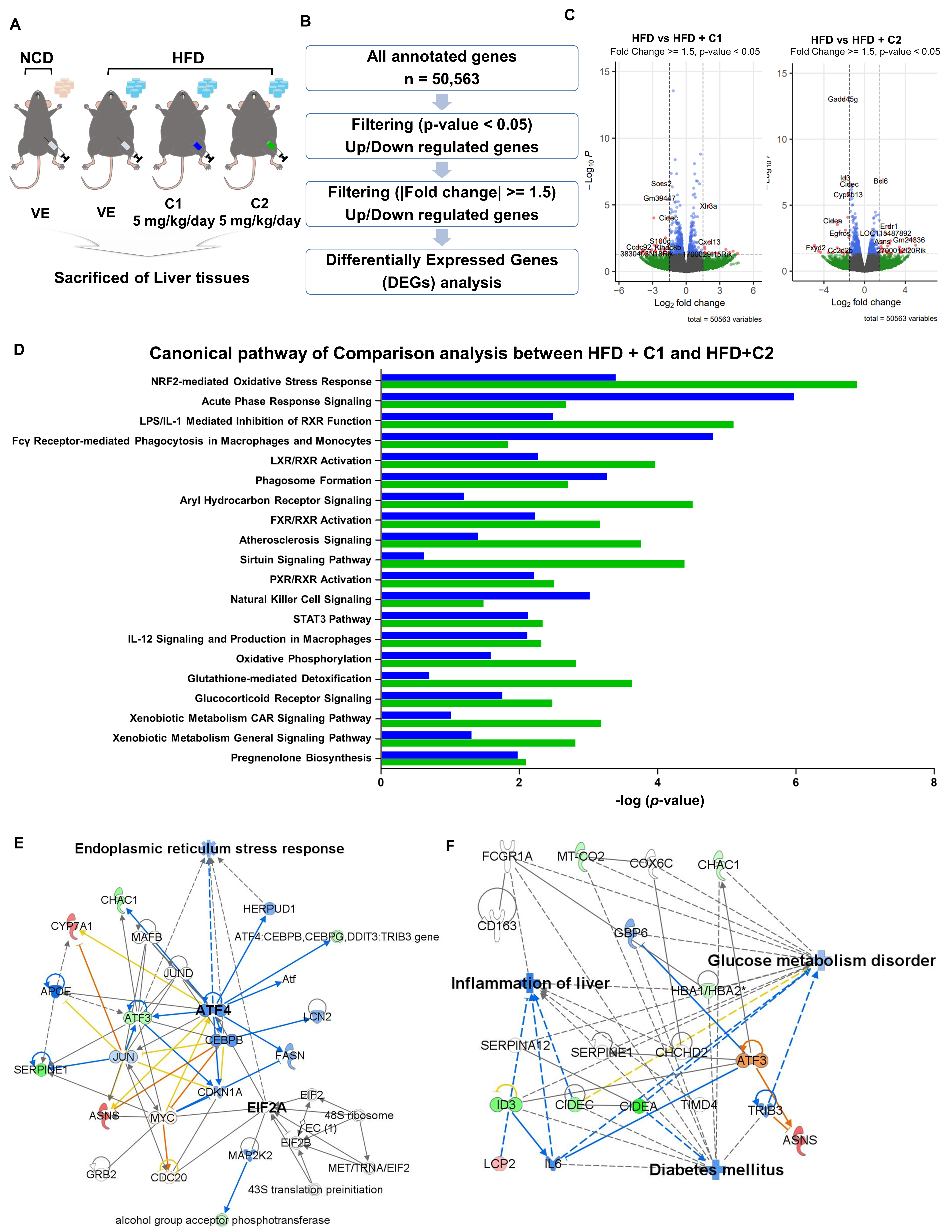


Figure S10 Overview of transcriptomic profiles of HFD-fed mice and HFD-fed mice treated with C1 or C2. (A) Scheme of RNA sequencing analysis. (B) Processing method of raw data. (C) Volcano plots for the HFD-fed mouse group compared with the HFD-fed group treated with each CYP4A inhibitor (C4181(C1) or C4182(C2)). Red dots represent the significant DEGs (|Fold Change| >= 1.5, *p*-value < 0.05). (D) Comparison analysis of canonical pathway between HFD + C1 and HFD+ C2. The comparison analysis shows C1 and C2 operate through fundamentally similar pathways. (E) Regulatory networks of C1 with overlapping DEGs data regulated by C2. (F) Regulatory networks of C2 with overlapping DEGs data regulated by C1. Red indicates upregulated genes, and green indicates downregulated genes. Orange represents the predicted activation of nodes, and blue represents the predicted inactivation of nodes.

**Reference**

1. Di L, Kerns EH, Hong Y, Chen H. Development and application of high throughput plasma stability assay for drug discovery. *International journal of pharmaceutics*. 2005;297(1-2):110-119.

2. Yong J, Parekh VS, Reilly SM, et al. Chop/Ddit3 depletion in β cells alleviates ER stress and corrects hepatic steatosis in mice. *Science translational medicine*. 2021;13(604):eaba9796.

3. Tan M, Mosaoa R, Graham GT, et al. Inhibition of the mitochondrial citrate carrier, Slc25a1, reverts steatosis, glucose intolerance, and inflammation in preclinical models of NAFLD/NASH. *Cell Death & Differentiation*. 2020;27(7):2143-2157.

4. Guo T, Yan W, Cui X, et al. Liraglutide attenuates type 2 diabetes mellitus-associated non-alcoholic fatty liver disease by activating AMPK/ACC signaling and inhibiting ferroptosis. *Molecular Medicine*. 2023;29(1):132.

5. Dong J, Viswanathan S, Adami E, et al. Hepatocyte-specific IL11 cis-signaling drives lipotoxicity and underlies the transition from NAFLD to NASH. *Nature communications*. 2021;12(1):66.

6. Yu J, Zhu H, Kindy MS, Taheri S. Cytochrome P450 CYP2E1 suppression ameliorates cerebral ischemia reperfusion injury. *Antioxidants*. 2021;10(1):52.

7. Bou-Fakhredin R, Dia B, Ghadieh HE, et al. CYP450 Mediates Reactive Oxygen Species Production in a Mouse Model of β-Thalassemia through an Increase in 20-HETE Activity. *International Journal of Molecular Sciences*. 2021;22(3):1106.

8. Tunaru S, Bonnavion R, Brandenburger I, et al. 20-HETE promotes glucose-stimulated insulin secretion in an autocrine manner through FFAR1. *Nature communications*. 2018;9(1):177.
